# Evaluation of SNP calling methods for closely related bacterial isolates and a novel high-accuracy pipeline: BactSNP

**DOI:** 10.1099/mgen.0.000261

**Published:** 2019-05-17

**Authors:** Dai Yoshimura, Rei Kajitani, Yasuhiro Gotoh, Katsuyuki Katahira, Miki Okuno, Yoshitoshi Ogura, Tetsuya Hayashi, Takehiko Itoh

**Affiliations:** ^1^​ School of Life Science and Technology, Tokyo Institute of Technology, Tokyo, Japan; ^2^​ Department of Bacteriology, Faculty of Medical Sciences, Kyushu University, Fukuoka, Japan

**Keywords:** SNP calling, bacterial comparative genomics, molecular epidemiology, benchmark, whole genome sequencing

## Abstract

Bacteria are highly diverse, even within a species; thus, there have been many studies which classify a single species into multiple types and analyze the genetic differences between them. Recently, the use of whole-genome sequencing (WGS) has been popular for these analyses, and the identification of single-nucleotide polymorphisms (SNPs) between isolates is the most basic analysis performed following WGS. The performance of SNP-calling methods therefore has a significant effect on the accuracy of downstream analyses, such as phylogenetic tree inference. In particular, when closely related isolates are analyzed, e.g. in outbreak investigations, some SNP callers tend to detect a high number of false-positive SNPs compared with the limited number of true SNPs among isolates. However, the performances of various SNP callers in such a situation have not been validated sufficiently. Here, we show the results of realistic benchmarks of commonly used SNP callers, revealing that some of them exhibit markedly low accuracy when target isolates are closely related. As an alternative, we developed a novel pipeline BactSNP, which utilizes both assembly and mapping information and is capable of highly accurate and sensitive SNP calling in a single step. BactSNP is also able to call SNPs among isolates when the reference genome is a draft one or even when the user does not input the reference genome. BactSNP is available at https://github.com/IEkAdN/BactSNP.

## Data Summary

BactSNP is available at https://github.com/IEkAdN/BactSNP and simulated correct SNPs and reads in the benchmarks are available at http://platanus.bio.titech.ac.jp/bactsnp.

**Impact Statement**

While a number of SNP-calling methods have been developed, their performance in calling SNPs among closely related bacterial isolates have not been validated sufficiently. This study represents realistic benchmarks to reveal that some of those methods exhibit low-accuracy results. As an alternative method, we developed a novel pipeline, BactSNP, which can detect SNPs both accurately and sensitively. BactSNP provides every researcher, even those lacking well-trained bioinformatic skills, a user-friendly tool to identify SNPs highly accurately, and will accelerate microbial genomic research.

## Introduction

While many studies on the intra-species genetic diversity of bacteria have been conducted, this research area is at a turning point in terms of molecular typing technologies. Whole-genome sequencing (WGS) is now often used instead of conventional methods such as multi-locus sequence typing (MLST) or pulsed-field gel electrophoresis (PFGE). WGS enables the differentiation of the genetic features of isolates, even when conventional methods cannot identify differences owing to the exceedingly high similarity between isolates [1, 2]. WGS is not only used to identify genetic variants causing phenotypic differences, but also to infer the infection routes of pathogenic bacteria in outbreaks where the target isolates are extremely closely related, sometimes indicating possible infection routes not identified by epidemiological data [2, 3].

In such WGS-based studies, the most basic analysis consists of identifying single-nucleotide polymorphisms (SNPs) among target isolates; these are variants that may explain phenotypes and provide the basis for phylogeny inference or other downstream analyses. Various SNP callers, including SAMtools [4], GATK [5], Freebayes [6], VarScan [7] and Cortex [8] have been developed and used in many comparative genomic studies of bacteria (e.g. SAMtools, [9, 10]; GATK, [11, 12]; Freebayes, [13, 14]; VarScan [15, 16], Cortex, [17, 18]). While SAMtools, GATK, Freebayes and VarScan are mapping-based tools that require the result of sequence-read mapping as their input, Cortex is a de Bruijn graph-based tool that detects SNPs by directly loading the reads of multiple samples into the same de Bruijn graph [8]. As WGS has become popular in bacterial comparative genomics, some dedicated pipelines, such as CFSAN SNP Pipeline (CFSAN) [19], NASP [20], PHEnix [21], and Snippy [22] have been developed for SNP calling among bacterial isolates. In general, these pipelines require the reads of target isolates and a reference genome as input and execute an external mapping tool and variant caller, followed by some filtering steps to remove low-quality SNPs.

In spite of the importance of SNP calling and the increasing number of SNP callers, the accuracy of SNP-calling methods has not been validated sufficiently. In particular, when researchers focus on closely related isolates, the accumulation of false-positive SNPs between each isolate and the reference isolate easily leads to a high number of false-positive SNPs compared with the limited number of true SNPs among isolates. To our knowledge, there have been no studies that have benchmarked the sensitivity and accuracy of SNP calling methods among bacterial isolates.

Here, we describe realistic benchmarks of these tools in calling SNPs among closely related isolates and reveal that some of them often exhibit low accuracy. In addition, we present a novel pipeline BactSNP. Though BactSNP, like the above-mentioned pipelines, also uses mapping information, it simultaneously *de novo* assembles the input reads and uses the alignment information between the assembled contigs and the reference genome to avoid false positives. Our benchmarks demonstrate that BactSNP achieves highly sensitive and accurate SNP calling.

## Methods

### Benchmarking of SNP callers

**Fig. 1.**
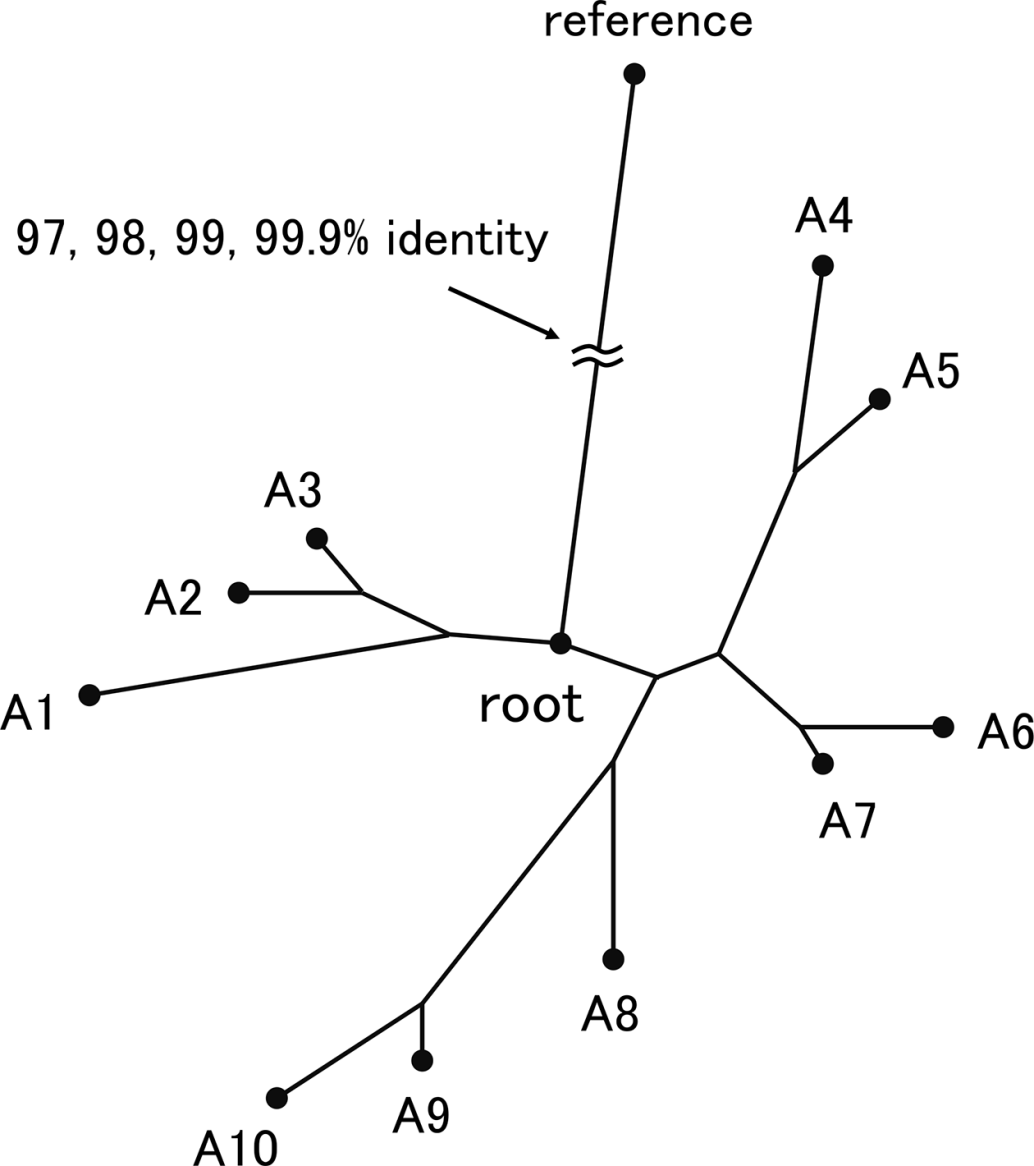
Conceptual diagram of analysis situation simulated in benchmark. Reference and root sequence pairs exhibiting approximately 97 %, 98 %, 99 % and 99.9 % identity were chosen from complete genomes in the NCBI database and the genomes of 10 virtual isolates and their reads were simulated.

When researchers call SNPs among isolates, they generally use a known reference genome, and SNPs are called at positions along this genome. We simulated this situation as shown in Fig. 1.

First, the complete genomes of *Staphylococcus aureus*, *Neisseria meningitidis* and *Escherichia coli* were downloaded from the NCBI database [23], and paired root and reference sequences exhibiting approximately 97, 98, 99 and 99.9 % identity were selected for each species (Table S1, available in the online version of this article) to simulate various cases. Next, for each reference-root pair, the genomes of 10 virtual isolates (A1–A10) were simulated by introducing variants to the root sequence. In order to reproduce the situation where target isolates are closely related, variants were introduced using EvolveAGene [24] so that each edge on the tree contains approximately 10 substitutions, 1 insertion and 0.25 deletions on average.

Simulated substitutions were moved to random positions in regions where nucmer [25] generated one-to-one alignments≥1 kbp in length between the pair sequences using our in-house program. This procedure was used so that we could check whether the detected SNP positions in the reference sequence corresponded to the true SNP positions simulated in the root sequence. Then, Illumina paired-end reads with sequencing errors were simulated from the genome of each virtual isolate using ART [26]. Finally, we called SNPs among isolates A1–A10 using the simulated reads and the reference genome with the above-mentioned SNP callers. See Supplementary Notes for a detailed description of this benchmark and the executed commands.

The advantage of this benchmark is that variants between the reference and the target isolates are not simulated but real, complicated ones, except for the substitutions introduced against the root to simulate each target isolate's sequence. These complicated variants make SNP calling realistically difficult, therefore this benchmark can precisely evaluate the accuracy of each tool.

## Results

### Benchmarking of general SNP callers

**Fig. 2.**
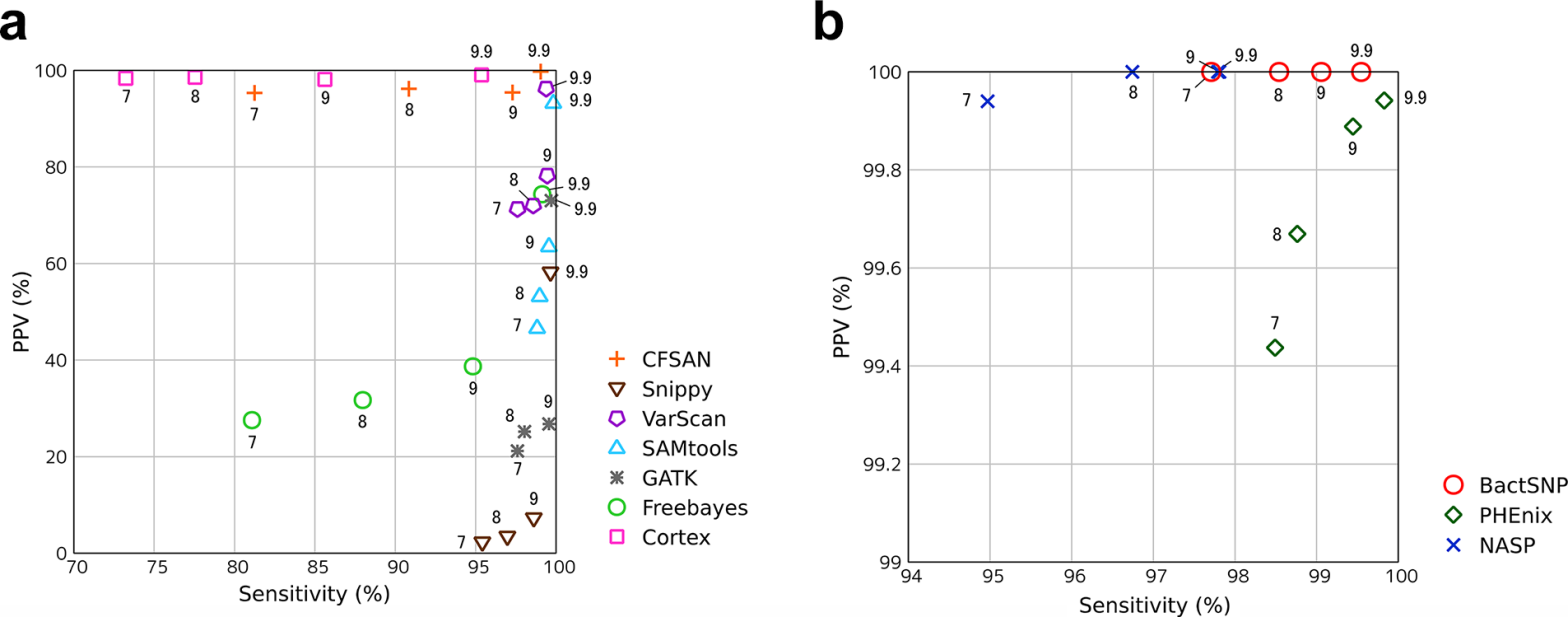
Benchmarks using simulated sequence data. PPV and Sensitivity in Table 1(a) were represented graphically (Those in Table 1(b) and (c) are represented in Fig. S1). The values 7, 8, 9 and 9.9 in the graph represent 97, 98, 99 and 99.9 % identity between the reference-root pair, respectively. (a) PPV and sensitivity of SNP callers that exhibited low PPV (<99) in at least one identity. (b) PPV and sensitivity of SNP callers that exhibited high PPV (≥99) in all identities.

First, we validated the above-mentioned general SNP callers, i.e., Cortex, Freebayes, GATK, SAMtools, and VarScan, which are not dedicated to calling SNPs among bacterial isolates (Table 1, Figs 2 and S1).

**Table 1.** Benchmarks using simulated sequence data Public complete genomes of three species, including (a) *S. aureus*, (b) *N. meningitidis* and (c) *E. coli*, were used for the benchmark. Identity, sequence identity between the root and reference isolates; positive predictive value (PPV), ratio of true-positive detected SNP sites to all detected SNP sites; Sensitivity, ratio of true-positive detected SNP sites to all true SNP sites; Called-sites, ratio of sites where a nucleotide was determined unambiguously in all isolates to the reference-genome size. All stats are averaged values among ten duplications. Called-sites ratio was not calculated for Cortex and CFSAN and was represented as ‘–’, because they do not output information for non-variant regions.

(a)	Identity (%)	Cortex	Freebayes	GATK	SAMtools	VarScan	Snippy	CFSAN	NASP	PHEnix	BactSNP
PPV (%)	99.9	99.07	74.35	73.04	93.36	96.27	58.05	99.78	100.00	99.94	100.00
	99	98.16	38.69	26.75	63.68	78.22	7.13	95.45	100.00	99.89	100.00
	98	98.59	31.71	25.18	53.33	72.04	3.31	96.22	100.00	99.67	100.00
	97	98.37	27.55	21.17	46.73	71.34	2.13	95.34	99.94	99.44	100.00
Common-region PPV (%)	99.9	100.00	100.00	98.79	99.91	100.00	94.24	100.00	100.00	100.00	100.00
	99	99.89	98.05	93.55	98.76	98.50	48.47	100.00	100.00	100.00	100.00
	98	100.00	98.46	95.34	97.63	99.48	33.57	100.00	100.00	100.00	100.00
	97	100.00	99.17	89.87	96.88	98.60	24.31	99.84	100.00	100.00	100.00
Sensitivity (%)	99.9	95.37	99.15	99.71	99.83	99.39	99.66	99.04	97.81	99.83	99.55
	99	85.63	94.83	99.56	99.56	99.45	98.62	97.29	97.79	99.45	99.06
	98	77.55	87.97	98.04	98.99	98.59	96.98	90.85	96.74	98.76	98.54
	97	73.24	81.09	97.60	98.82	97.60	95.42	81.25	94.97	98.49	97.71
Called-sites (%)	99.9	–	88.92	95.36	64.40	95.96	94.19	–	91.91	94.78	93.69
	99	–	84.31	90.11	61.24	89.88	87.80	–	87.16	89.37	88.25
	98	–	84.96	90.40	61.86	89.97	86.69	–	87.27	89.42	88.30
	97	–	84.38	89.65	61.71	88.90	84.66	–	86.39	88.39	87.12

(b)	Identity (%)	Cortex	Freebayes	GATK	SAMtools	VarScan	Snippy	CFSAN	NASP	PHEnix	BactSNP
PPV (%)	99.9	96.08	94.94	96.21	98.61	98.91	47.46	99.56	100.00	100.00	100.00
	99	96.38	50.35	41.42	71.33	80.49	5.77	96.10	100.00	99.66	100.00
	98	96.57	27.24	19.41	46.44	61.13	2.72	91.34	99.83	99.89	100.00
	97	96.81	23.68	16.88	43.20	64.76	2.12	91.29	100.00	99.66	100.00
Common-region PPV (%)	99.9	99.38	100.00	99.89	99.89	100.00	97.47	100.00	100.00	100.00	100.00
	99	99.41	99.44	98.04	99.18	99.79	81.17	99.78	100.00	100.00	100.00
	98	99.35	98.64	93.38	98.11	99.67	69.99	99.38	100.00	100.00	100.00
	97	99.51	98.67	89.41	98.14	99.74	62.64	99.70	100.00	100.00	100.00
Sensitivity (%)	99.9	95.43	99.16	99.55	99.49	99.33	98.61	97.33	96.09	98.94	97.32
	99	89.44	94.30	99.17	98.95	98.89	98.17	93.26	96.30	98.78	98.01
	98	82.86	88.95	98.41	98.69	97.97	96.04	84.91	95.43	98.30	97.36
	97	74.35	84.05	97.54	97.70	96.53	94.26	76.42	94.40	97.65	96.65
Called-sites (%)	99.9	–	91.19	97.74	66.25	99.30	95.21	–	88.05	96.47	91.41
	99	–	88.64	94.50	64.33	96.14	90.67	–	85.89	92.95	89.20
	98	–	86.79	91.86	63.25	92.30	85.30	–	82.38	89.29	85.60
	97	–	81.61	86.06	59.54	86.04	78.85	–	78.48	83.62	80.90

(c)	Identity (%)	Cortex	Freebayes	GATK	SAMtools	VarScan	Snippy	CFSAN	NASP	PHEnix	BactSNP
PPV (%)	99.9	98.60	35.81	35.04	89.98	83.32	56.27	99.89	100.00	100.00	100.00
	99	98.43	28.60	21.72	74.45	76.59	5.38	96.78	100.00	99.94	100.00
	98	98.70	15.03	9.95	42.77	52.34	2.39	94.90	100.00	99.78	100.00
	97	99.05	23.64	18.70	70.21	81.90	1.54	94.24	99.94	99.83	100.00
Common-region PPV (%)	99.9	100.00	99.30	94.66	99.89	99.89	96.48	100.00	100.00	100.00	100.00
	99	99.76	98.76	89.64	99.48	99.78	76.46	99.76	100.00	100.00	100.00
	98	100.00	96.56	78.04	98.63	98.84	54.01	99.74	100.00	100.00	100.00
	97	100.00	97.35	84.23	99.31	99.86	52.38	98.98	100.00	100.00	100.00
Sensitivity (%)	99.9	97.47	99.22	99.72	99.67	99.50	99.33	98.39	98.78	99.50	99.33
	99	93.67	95.12	99.66	99.49	98.87	98.48	97.08	98.09	99.61	99.16
	98	87.30	88.01	98.95	99.17	98.73	98.21	91.78	97.10	99.33	99.00
	97	83.21	78.37	98.70	99.21	98.20	96.79	80.94	95.31	98.93	98.81
Called-sites (%)	99.9	–	88.78	95.09	64.12	96.55	93.01	–	90.19	94.02	92.40
	99	–	82.60	88.20	59.80	88.39	86.07	–	85.56	87.39	86.44
	98	–	78.74	83.60	56.95	83.49	79.77	–	79.59	82.19	81.00
	97	–	74.93	79.52	54.62	80.10	76.24	–	77.28	78.68	77.89

Surprisingly, with the exception of Cortex, they exhibited low positive predictive values (PPVs), even though their results were filtered following the respective manuals. It is worth noting that these tools exhibited low PPVs (<90) even when the identity between the reference and the target isolates was relatively high. Cortex showed a PPV ≥90 for all cases, but its sensitivity was relatively low, especially in the case of low identity. This benchmark tends to overestimate sensitivity because SNPs among isolates were simulated only in regions where SNP calling should be easy (i.e. a one-to-one alignment was generated between the reference and the root isolate), indicating that the sensitivity of Cortex in real analyses would be even lower.

To overcome this overestimation problem, as an alternative index of sensitivity, we calculated the called-sites ratio which is the ratio of sites where the nucleotide was determined unambiguously in all isolates to the genome size. In the case where a phylogenetic tree is inferred with complete deletion (i.e. sites containing missing data or alignment gaps are discarded), the called-sites ratio determines the number of SNPs used in the phylogenetic analysis. This index revealed that SAMtools would be less sensitive in real analyses.

The common feature of the low-accuracy general SNP callers is that they use mapping information. In order to reveal the reason for these low-accuracy results, we checked how reads were mapped around the false-positive SNPs. The majority of false-positive SNPs turned out to be located in ‘soft-clip regions’ where many reads were soft-clipped (i.e. a read was partially not aligned because it was difficult to align the whole read to a single region) (Table 2, Fig. S2). It is considered that such dense soft-clipping is caused by structural variants (SVs), e.g. copy number variations (Fig. S3). PPVs could be increased by masking ‘soft-clip regions’, but sensitivities significantly decreased (Table S3). We also tried removing soft-clipped reads from the input bam files, but the performance did not improve (Table S4). In contrast, Cortex is designed to filter false positives related to copy number variations; it loads the reads of multiple isolates into the same de Bruijn graph and detects variants as bubble structures in the graph; it then filters bubbles that are present in all target isolates or the reference isolate as repeat-induced bubbles [8].

**Table 2.** Ratio of false-positive SNP sites in ‘soft-clip regions’ to all false-positive SNP sites Public complete genomes of three species including (a) *S. aureus*, (b) *N. meningitidis* and (c) *E. coli* were used for the benchmarks. The definition of ‘soft-clip region’ is described in Fig. S2. In the middle nine columns, the numerator of the fraction denotes the number of false-positive SNP sites in ‘soft-clip regions’; the denominator, the number of all false-positive SNP sites; the number in parentheses, the value of the fraction. In the rightmost column, the numerator of the fraction denotes the total size of ‘soft-clip regions’; the denominator, the reference-genome size; the value in parentheses, the value of the fraction. This table is based on the results for the first reference-root pair among ten pairs in each species and identity (Table S1). Results for BactSNP were not shown, because they did not detect any false-positives.

(a)	Identity (%)	Cortex	Freebayes	GATK	SAMtools	VarScan	Snippy	CFSAN	PHEnix	NASP	Soft-clip region
	99.9	0/0	31/33	40/42	3/3	1/1	143/222	2/2	0/0	0/0	62,258/2,824,404
		(–)	(93.00)	(95.00)	(100.00)	(100.00)	(64.00)	(100.00)	(–)	(–)	(2.20)
	99	0/6	264/280	497/514	70/73	35/35	1,739/2198	6/6	0/0	0/0	282,639/2,742,807
		(0.00)	(94.00)	(96.00)	(95.00)	(100.00)	(79.00)	(100.00)	(–)	(–)	(10.30)
	98	0/0	380/402	602/624	127/139	46/52	4,543/5262	4/4	0/0	0/0	545,715/3,046,545
		(–)	(94.00)	(96.00)	(91.00)	(88.00)	(86.00)	(100.00)	(–)	(–)	(17.91)
	97	2/8	226/233	466/475	125/129	49/49	7,211/8146	8/9	3/3	0/0	754,175/2,778,079
		(25.00)	(96.00)	(98.00)	(96.00)	(100.00)	(88.00)	(88.00)	(100.00)	(–)	(27.14)

(b)	Identity (%)	Cortex	Freebayes	GATK	SAMtools	VarScan	Snippy	CFSAN	PHEnix	NASP	Soft-clip region
	99.9	0/8	5/8	2/4	0/0	0/0	253/263	1/1	0/0	0/0	46,468/2,162,199
		(0.00)	(62.00)	(50.00)	(–)	(–)	(96.00)	(100.00)	(–)	(–)	(2.14)
	99	0/4	146/179	199/244	43/58	23/30	3,258/3432	12/12	0/0	0/0	393,578/2,188,020
		(0.00)	(81.00)	(81.00)	(74.00)	(76.00)	(94.00)	(100.00)	(–)	(–)	(17.98)
	98	0/10	292/344	491/557	91/116	40/50	5,525/6008	9/9	0/0	0/0	701,290/2,273,677
		(0.00)	(84.00)	(88.00)	(78.00)	(80.00)	(91.00)	(100.00)	(–)	(–)	(30.84)
	97	1/3	378/445	715/783	222/252	89/99	7,667/8191	12/13	1/1	0/0	888,197/2,188,020
		(33.00)	(84.00)	(91.00)	(88.00)	(89.00)	(93.00)	(92.00)	(100.00)	(–)	(40.59)

(c)	Identity (%)	Cortex	Freebayes	GATK	SAMtools	VarScan	Snippy	CFSAN	PHEnix	NASP	Soft-clip region
	99.9	0/3	185/271	235/323	10/22	36/57	128/136	0/0	0/0	0/0	123,933/5,438,591
		(0.00)	(68.00)	(72.00)	(45.00)	(63.00)	(94.00)	(–)	(–)	(–)	(2.27)
	99	0/4	161/211	184/246	10/27	12/20	2,443/3163	2/2	0/0	0/0	372,887/4,682,086
		(0.00)	(76.00)	(74.00)	(37.00)	(60.00)	(77.00)	(100.00)	(–)	(–)	(7.96)
	98	0/3	744/918	1,376/1593	183/234	136/170	7,624/9419	6/6	1/1	0/0	957,794/5,310,511
		(0.00)	(81.00)	(86.00)	(78.00)	(80.00)	(80.00)	(100.00)	(100.00)	(–)	(18.03)
	97	0/1	358/393	747/785	68/72	18/23	9,227/11190	14/14	0/0	0/1	1,087,260/4,658,583
		(0.00)	(91.00)	(95.00)	(94.00)	(78.00)	(82.00)	(100.00)	(–)	(0.00)	(23.33)

PPVs occasionally did not show monotonic decline with identity, and this is assumed to be an effect of SVs and repetitive regions where SNP calling is difficult. Identity was calculated using substitutions in one-to-one alignment regions, but the frequency of SVs and repetitive regions does not necessarily increase monotonically, and one SV or repetitive region sometimes causes a large number of dense false positives.

SNP callers often mask regions where it is difficult to call SNPs correctly. Importantly, the larger the masked region, the easier it becomes to achieve high PPVs. In order to compare PPVs among tools that mask some regions in different ways, we introduced ‘Common-region PPV’, i.e. PPV calculated only in regions where all tools determined alleles for all isolates without masking (Tables 1 and S5). Because it should be easy to correctly call SNPs in such a region, the Common-region PPVs were much higher than the raw PPVs; however, GATK exhibited relatively low Common-region PPVs.

We also carried out another supplementary benchmark in which variants between the reference and the target isolates were simulated by TreeToReads [27] (Supplementary Notes). General mapping-based SNP callers exhibited higher PPVs in this benchmark than in the first benchmark (Table S6). Considering that TreeToReads does not simulate SVs, this result indicates that real, complex variants between the reference and root sequence used in the first benchmark surely caused many false-positive SNP calls and enabled realistic evaluation of the accuracy.

### Benchmarking of dedicated SNP calling pipelines

We validated CFSAN, NASP, PHEnix and Snippy as SNP calling pipelines dedicated for use with multiple bacterial isolates.

CFSAN, NASP and PHEnix achieved higher PPVs compared with their internal variant callers (CFSAN used VarScan, and NASP and PHEnix used GATK; Supplementary Notes). These pipelines filter ambiguous SNPs with low coverage depth or low allele frequency, and thus these filters are considered to be effective against false positives called by VarScan and GATK (Supplementary Notes). CFSAN still called a relatively large number of false positives, though it additionally filters high-density SNPs; therefore, further filtering procedures may be required for VarScan.

In contrast, Snippy exhibited low PPVs though it also filtered SNPs with low coverage depth or allele frequency, indicating that the internal variant caller, Freebayes, called many false positives that were not filtered by these criteria.

### Development of BactSNP

Although PHEnix exhibited high PPVs, it often called a few false positives. NASP did not call any false positives in most cases; however, it exhibited lower sensitivities than PHEnix. Regarding usability, when the reference genome was a draft one, the vcf2fasta command of PHEnix, which converts the VCF files of multiple isolates into a multi-FASTA file to obtain SNPs among them, did not work. We developed a novel assembly-based pipeline, BactSNP, and verified whether it settled these problems. BactSNP also uses the mapping information secondarily, but it is mainly based on the alignment information between the reference genome and the contigs of the target isolates which are *de novo* assembled internally.

BactSNP uses reads of each isolate and a reference genome as input. First, the reads are *de novo* assembled by Platanus [28] for each isolate, and then the assembled contigs are aligned against the reference genome by nucmer. Second, the nucleotide corresponding to the reference genome at each site is determined to generate the ‘pseudogenome’ (i.e. a sequence in which each site corresponds to the reference genome in one-to-one manner) for each isolate. The variables *n_allele_* and *d_indel_* represent the number of alleles aligned at the site and the distance from the nearest indel to the site, respectively. At each site in the reference genome, the corresponding allele is determined as the aligned allele if the site satisfies both of the following conditions:

(1)*n_allele_*=1

(2)*d_indel_* >5 bp

In addition to assembling the reads, they are mapped to the reference genome by BWA-MEM [29] and duplicate reads generated by PCR duplication are removed by MarkDuplicates [30]. By using the mapping information, unreliable alleles of the pseudogenome are masked. The variables *c_all_* and *c_allele_* represent the coverage depth of all reads mapped to the site and that of the reads supporting the allele of the pseudogenome at the site, respectively. The corresponding allele of the pseudogenome is masked if the reference site does not satisfy either of the following conditions:

(3)*c_allele_* ≥10

(4)*c_allele_*/*c_all_*≥0.9

Lastly, SNPs among isolates are determined by using the pseudogenomes generated in one-to-one manner.

BactSNP works well, even when the reference genome is a draft one. A detailed description of the algorithm is provided in the Supplementary Notes.

### Benchmarking of BactSNP

We evaluated BactSNP using the above-mentioned benchmark (Table 1). While PHEnix often called some false positives, BactSNP did not detect even one false-positive SNPs among all cases. In addition, it achieved higher sensitivities and a larger number of called-sites than NASP in all cases.

### Application to real data

**Fig. 3.**
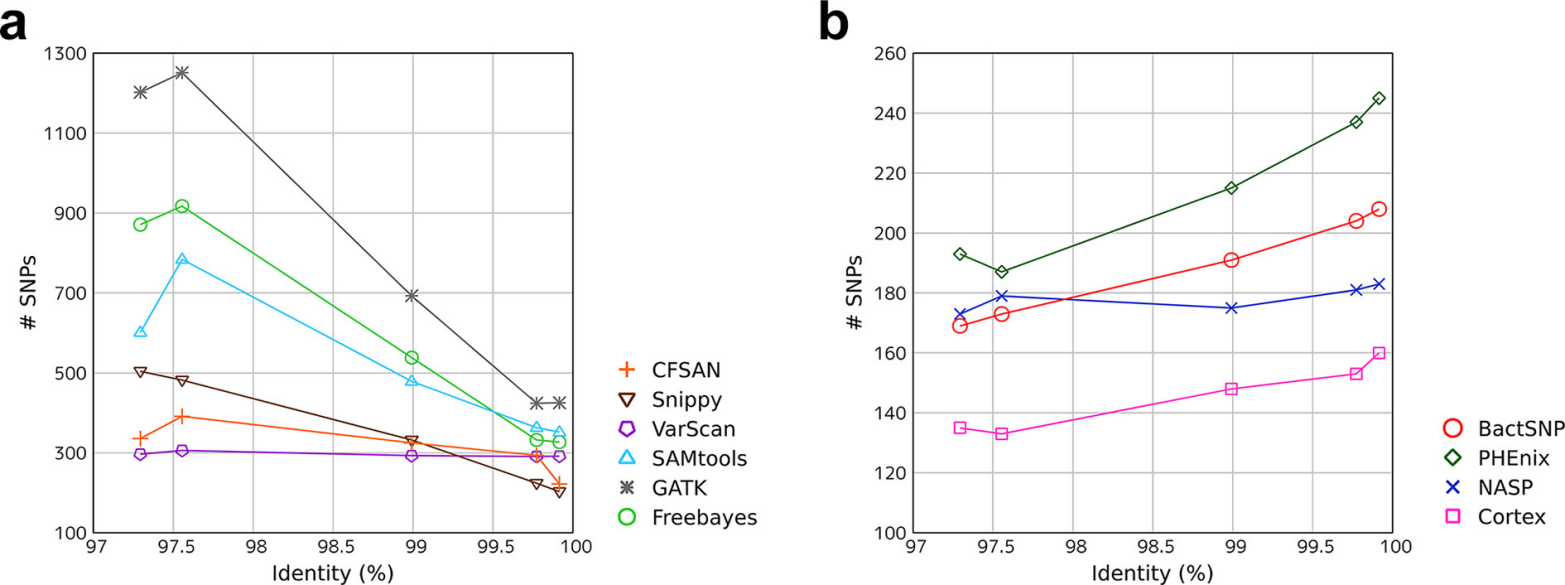
Number of detected SNP sites in real sequence data analysis. The relationship between the identity between the reference isolate and the target isolates and the number of detected SNP sites among the target isolates is shown. (a) SNP callers that exhibited relatively low PPV (<95) in at least one identity. (b) SNP callers that exhibited relatively high PPV (≥95) in all identities.

In order to validate the performance of these SNP callers in real data analysis, we applied them to the read data sequenced in a comparative genomic study on *N. meningitidis* [31]. We downloaded the sequence data of 45 closely-related isolates which caused outbreaks in Ghana (Supplementary Notes). Because the true SNPs cannot be known, we compared the number of detected SNP sites among target isolates when multiple reference genomes were used. Basically, the number of detected SNP sites should be at most constant and will probably slightly decrease in the low-identity cases, as the common region between the target isolates and the reference genome gets smaller. We tested five reference genomes with various identities from the target isolates (Supplementary Notes) and obtained results consistent with the benchmark (Fig. 3, Table S7). SNP callers that exhibited low PPVs in the benchmark tended to detect more SNPs in the low-identity cases, probably due to an increase in false-positive SNPs. In contrast, tools with high PPVs in the benchmark tended to call a smaller number of SNPs in the low-identity cases.

## Discussion

Our benchmark revealed that some SNP callers detect a large number of false positives. In contrast, some dedicated pipelines including NASP, PHEnix, and our novel pipeline BactSNP achieved both highly accurate and sensitive SNP calling, regardless of the identity between the reference and target isolates. Therefore, these pipelines are powerful even when a reliable and well-annotated reference genome closely related to the target isolates is not available or when a subset of target isolates is not closely related to the others and the user cannot therefore use a reference genome close to all of them.

BactSNP did not call even one false positive, while PHEnix often called some false positives. The sensitivity of BactSNP was slightly lower than that of PHEnix, but it exceeded that of NASP in all cases involving various species and identities.

As for usability, the filtering parameters of BactSNP are well optimized and set as the default, whereas the user is required to consider the parameters or even the internal mapper and variant caller in the other pipelines. Unlike PHEnix, BactSNP is also usable when the reference genome is a draft one. In addition, BactSNP can be used even when the user does not specify a reference genome; in this case, it *de novo* assembles one of the target isolates, which the user can specify, and uses it as the reference genome automatically. This function is useful when the user only needs the phylogeny of the target isolates and does not require the SNP position to be well annotated in the reference genome. In this case, the assembled reference genome, which is exceedingly closely related to the other target isolates in outbreak studies, would enable highly sensitive SNP calling, and the user does not need to consider the proper reference genome. BactSNP creates a TSV file containing SNP information and an alignment FASTA file containing the constructed pseudogenomes of target isolates in a single step. The alignment FASTA file can be input to Gubbins [32] to predict recombination regions containing a statistically elevated density of SNPs and reconstruct a phylogenetic tree using SNPs outside those regions.

BactSNP is expected to enable every researcher, even those who do not have proficient bioinformatic skills, to obtain accurate SNP information easily and to aid and accelerate microbial genomic research.

## Data bibliography

1. BactSNP is available at https://github.com/IEkAdN/BactSNP and simulated correct SNPs and reads in the benchmarks are available at http://platanus.bio.titech.ac.jp/bactsnp.

## Supplementary Data

Supplementary File 1
