# Supplementary material for: Evaluation of SNP calling methods for closely related bacterial isolates and a novel high-accuracy pipeline: BactSNP
Source: Microb Genom. 2019 May 17;5(5):e000261. doi: 10.1099/mgen.0.000261 (PMC6562250; doi:10.1099/mgen.0.000261)
Supplement: Supplementary File 1 [file mgen-5-261-s001.pdf]

Supplementary Material

Article Title

Evaluation of SNP calling methods for closely related bacterial isolates and a novel high-accuracy pipeline: BactSNP

Authors

Dai Yoshimura[1], Rei Kajitani[1], Yasuhiro Gotoh[2], Katsuyuki Katahira[2], Miki Okuno[1], Yoshitoshi Ogura[2], Tetsuya Hayashi[2], Takehiko Itoh[1]

[1]School of Life Science and Technology, Tokyo Institute of Technology, Meguro-ku, Tokyo 152-8550, Japan.
[2]Department of Bacteriology, Faculty of Medical Sciences, Kyushu University, 3-1-1 Maidashi, Higashi-ku, Fukuoka 812-8582, Japan.

Corresponding author. Takehiko Itoh, School of Life Science and Technology, Tokyo Institute of Technology, Meguro-ku, Tokyo 152-8550, Japan. Tel.:81 3 5734 3430; E-mail: takehiko@bio.titech.ac.jp

Supplementary Notes

Benchmark using real complete genome pairs

For each identity case, 10 pairs of the root and reference sequences with proper identity were randomly chosen from the completed genomes from the NCBI database [1] (Table S1). The identity was calculated using MUMmer [2] as average identity in one-to-one alignment regions. The commands were as follows:

1. nucmer [a.fasta] [b.fasta]
2. delta_filter -1 [out.delta] >[filtered.delta]
3. show-coords -THrcl [filtered.delta] >[filtered.coords]
4. awk '{product_sum += ($5 * $7); len += $5} END {print product_sum / len}' [filtered.coords] >[out.identity]

delta-filter extracts one-to-one alignment by the option -1. 5th and the 7th columns in the .coords file represent the alignment identity and alignment length, respectively.

Next, for each reference-root pair, the genomes of 10 virtual isolates were simulated by introducing variants to the root sequence using EvolveAGene [3]. Executed commands were as follows:

1. mean_branch_len=$(echo "scale=7; 10 / <root_genome_size> + 0.0000001" | bc -l)
2. evolveagene -f [root_genome.txt] -t ran -n 10 -b $mean_branch_len -i 0.1 -d 0.025

In order to simulate the situation where target isolates are closely related, EvolveAGene was executed so that about 10 substitutions were generated per branch. Since the target number of substitutions was exceedingly small compared to the genome size and the number of simulated substitutions depended on random numbers (the random seed could not be specified), the resulting average number of substitutions, *s*, often deviated from 10. Therefore, we repeated EvolveAGene until the following inequality was satisfied.

$$9.5 \leq s \leq 10.5$$

Indels were also simulated by the option *-i* and *-d* with the default values, which represent the relative frequency of insertion and deletion against substitutions, respectively. EvolveAGene only accepts a root sequence whose length is a multiple of 3 considering the codon position, and thus, the last one or two bases were deleted before input when the root genome size was not a multiple of 3.

Though SNP callers detect SNPs on the position of reference genome, the true SNP positions are simulated on the root genome by EvolveAGene. In order to check whether the detected SNP positions in the reference sequence correspond to the correct SNP positions simulated in the root sequence, the correct SNP positions needed to be

moved to the region where the root and reference sequences were aligned in a one-to-one manner. Therefore, we moved the correct SNP positions to random positions in regions where nucmer generated one-to-one alignments ≥ 1 kbp in length between the reference-root sequences and edited the simulated genomes so that they had SNPs on the new positions by using our original program, move_snp (available at https://github.com/IEkAdN/BactSNP/tree/master/benchmark). Executed commands were as follows:

1. nucmer -p nucmer [root.fasta] [reference.fasta]
2. move_snp [prefix_Internal_Nodes_True_alignment.FASTA] [prefix_True_alignment.FASTA] [nucmer.delta] [reference.fasta] [root.fasta] [output_directory] | sort -nk2,2 >[correct_snp.tsv]

[prefix_Internal_Nodes_True_alignment.FASTA] and [prefix_True_alignment.FASTA] are multi-fasta files containing alignment of simulated genomes output by EvolveAGene. The output [correct_snp.tsv] contains the correct SNPs on the corresponding position of reference genome.

Then, illumina paired-end reads for every simulated genome were simulated by art_illumina [4] using the following commands:

art_illumina -i [genome.fa] -l 250 -f 40 -ss MS -m 500 -s 50 -rs <random_seed>

The options *-l*, *-f*, *-ss*, *-m*, *-s*, and *-rs* specified the read length, coverage depth, illumina sequencing system (MS represents MiSeq), mean size of the DNA fragment, standard deviation of the DNA fragment size, and random seed, respectively.

At last, SNP callers were executed using the simulated reads and the reference genome. Then, it was checked whether the detected SNPs matched with the simulated correct SNPs.

Simulated correct SNPs and reads are available at http://platanus.bio.titech.ac.jp/bactsnp.

Common-region PPV

In order to compare PPVs among tools which mask the region where SNP calling is difficult in different ways, we introduced 'Common-region PPV', i.e., PPV calculated based on the SNPs detected in the region where all tools except Cortex [5] and CFSAN SNP Pipeline (CFSAN) [6] determined alleles for all isolates without masking. Because Cortex and CFSAN were not able to output information for non-variant regions, they were not considered when calculating Common-region PPV. The average ratio of the common region to the reference genome size is shown in Table S5.

Execution of SNP callers

Before executing each tool, low-quality regions were trimmed from reads by Platanus_trim

(version 1.0.7) [7], and mapping was performed by BWA-MEM (version 0.7.15) [8] for tools that require mapping results as input. Duplicate reads generated by PCR duplication were removed by Picard MarkDuplicates (version 2.18.17) [9]. The commands were as follows:

1. platanus_trim [R1.fastq] [R2.fastq]
2. bwa index [reference.fa]
3. bwa mem -M [reference.fa] [R1_trimmed.fastq] [R2_trimmed.fastq] | samtools fixmate - - | samtools sort -o [raw.bam]
4. java -jar picard.jar MarkDuplicates REMOVE_DUPLICATES=true M=[out.log] I=[raw.bam] O=[markduplicates.bam]

(a) Cortex
run_calls.pl script in Cortex (version 1.0.5.21) was executed as described in the section "Comparing two strains of microbe" in its user manual (http://cortexassembler.sourceforge.net/). The hash table required for run_calls.pl was generated by Stampy [10]. SNPs which passed the filters in run_calls.pl and marked as "PASS" were extracted from the resulting .vcf file and output to a .tsv file using our original program, get_snp_cortex (available at https://github.com/IEkAdN/BactSNP/tree/master/benchmark). The commands were as follows:

1. stampy -G <stampy_prefix> [reference.fasta]
2. stampy -g <stampy_prefix> -H <stampy_prefix>
3. echo [reference.fasta] >[fasta_list]
4. make NUM_COLS=1 MAXK=31 cortex_var
5. make NUM_COLS=1 MAXK=63 cortex_var
6. cortex_var_31_c1 --kmer_size 31 --mem_height 17 --mem_width 100 --se_list [fasta_list] --dump_binary k31.ctx --sample_id REF
7. cortex_var_63_c1 --kmer_size 61 --mem_height 17 --mem_width 100 --se_list [fasta_list] --dump_binary k61.ctx --sample_id REF
8. echo [reference.fasta] >[reference.fasta_list]
9. run_calls.pl --first_kmer 31 --last_kmer 61 --kmer_step 30 --fastaq_index [fastq_list] --auto_cleaning yes --bc yes --pd no --outdir [output_directory] --outvcf out --ploidy 1 --stampy_hash <stampy_prefix> --stampy_bin stampy --list_ref_fasta [reference.fasta_list] --refbindir [reference_directory] --genome_size <genome_size> --qthresh 5 --mem_height 20 --mem_width 100 --vcftools_dir [vcftools_directory] --do_union yes --ref CoordinatesOnly --workflow joint --logfile 01.log --apply_pop_classifier
10. get_snp_cortex

[output_directory/vcfs/out_wk_flow_J_RefCO_FINALcombined_BC_calls_at_all_k.decomp.vcf] >[snp.tsv]

(b) Freebayes
Freebayes (version v1.2.0-2-g29c4002) [11] was executed as follows:

1. freebayes --ploidy 1 --report-monomorphic -f [reference.fasta] -L [markduplicates.bam_list] >[raw.vcf]
2. vcffilter -f 'QUAL > 20' -f 'TYPE = snp' [raw.vcf] >[filtered.vcf]
3. get_snp_freebayes [filtered.vcf] >[snp.tsv]

SNPs with QUAL > 20 were extracted by vcffilter as described in its README [12] and they were output to a .tsv file using our original program, get_snp_freebayes (available at https://github.com/IEkAdN/BactSNP/tree/master/benchmark).

(c) GATK
GATK HaplotypeCaller (version 4.0.11.0) [13] and the filtering steps were executed based on GATK Best Practices [14]. The base quality score recalibration and variant quality score recalibration steps were not executed because there were no available SNP databases for the isolates. Instead, we executed hard-filtering using VariantFiltration as described in the document. SNPs which passed the filter were extracted and output to a .tsv file using SelectVariants and our original program, get_snp_gatk (available at https://github.com/IEkAdN/BactSNP/tree/master/benchmark). GenotypeGVCFs in GATK version 4.0.11.0 has no option to emit information for all sites, and thus we used version 3.8-0-ge9d806836 for GenotypeGVCFs. The commands were as follows:

1. gatk HaplotypeCaller -ERC GVCF -ploidy 1 -R [reference.fasta] -I [markduplicates.bam] -O [out.vcf]
2. java -jar GenomeAnalysisTK.jar -T GenotypeGVCFs -allSites -R [reference.fasta] -V [A1.vcf] -V [A2.vcf] … -V [A10.vcf] -o [merged.vcf]
3. gatk VariantFiltration -filter-expression "QD < 2.0 || MQ < 40.0 || FS > 60.0 || SOR > 4.0 || MQRankSum < -12.5 || ReadPosRankSum < -8.0" --filter-name HARD_FLT -R [reference.fasta] -V [merged.vcf] -O [filtered.vcf]
4. gatk SelectVariants --select-type-to-include SNP -R [reference.fa] -V [filtered.vcf] -O [snp.vcf]
5. get_snp_gatk [snp.vcf] >[snp.tsv]

(d) SAMtools
SAMtools (version 1.9) [15] was executed based on the SAMtools "Workflows" document [16] and the method described in a previous study [17], which has been referred to in many studies that used SAMtools for SNP calling [18-20]. After mapped reads were

realigned by GATK IndelRealigner, PCR-duplicated reads were removed by MarkDuplicates and reads with mapping quality below 30 were eliminated. The base quality score recalibration step shown in the "Workflows" document was not executed because there are no available SNP databases for the isolates. Filtered reads were piled up by samtools mpileup, and the result was used as input for the bcftools call command. Called alleles at each site with a QUAL score below 30 or supported by fewer than 75% of reads mapped at that site were masked as ambiguous and the remaining SNPs were output to a .tsv file using our original program, get_snp_samtools (available at https://github.com/IEkAdN/BactSNP/tree/master/benchmark). The commands were as follows:

1. java -jar GenomeAnalysisTK.jar -T RealignerTargetCreator -R [reference.fa] -I [raw.bam] -o [out.intervals]
2. java -jar GenomeAnalysisTK.jar -T IndelRealigner -R [reference.fa] -I [raw.bam] -targetIntervals [out.intervals] -o [realigned.bam]
3. java -jar picard.jar MarkDuplicates M=[out.log] I=[realigned.bam] O=[out.bam]
4. samtools view -h [out.bam] | awk '$1 ~ /^@/ || ($1 !~ /^@/ && $5 >= 30)' | samtools view -b >[filtered.bam]
5. samtools index [filtered.bam]
6. bcftools mpileup -O u -f [reference.fa] [filtered.bam] | bcftools call --ploidy 1 -m -O v -o [isolate.vcf]
7. for isolate in $(seq 1 1 10); do echo "${isolate}\t[isolate.vcf]"; done >[out.vcf_list]
8. get_snp_samtools [out.vcf_list] >[snp.tsv]

(e) VarScan
The output of samtools (version 1.9) mpileup was input to VarScan (version 2.4.3) mpileup2cns [21]. SNPs among isolates shown in the output of mpileup2cns were extracted and output to a .tsv file using our original program, get_snp_varscan (available at https://github.com/IEkAdN/BactSNP/tree/master/benchmark) The commands were as follows:

1. samtools mpileup -f [reference.fa] -b [markduplicates.bam_list] >[out.mpileup]
2. java -jar VarScan.jar mpileup2cns [out.mpileup] -vcf-sample-list [isolate_list] >[out]
3. get_snp_varscan [out] >[snp.tsv]

(f) Snippy
The script generated by snippy-multi in Snippy (version 4.3.6) [22] was executed and SNPs among target isolates were extracted from the output core.tab using our original

program, get_snp_snippy (available at https://github.com/IEkAdN/BactSNP/tree/master/benchmark).

1. snippy-multi [fastq_list] --ref [reference.fasta] --minfrac 0.9 >[script.sh]
2. bash [script.sh]
3. get_snp_snippy <(cut --complement -f 3 [core.tab]) >[snp.tsv]

(g) CFSAN SNP Pipeline

CFSAN SNP Pipeline (version 2.0.2, CFSAN) masks high-density SNPs with the options "*--window_size 1000 125 15 --max_snp 3 2 1*" by default. These parameters mean that CFSAN filters more than 3 SNPs in 1000 bases, more than 2 SNPs in 125 bases and more than 1 SNPs in 15 bases. This default behavior assumes that the reference genome is closely related to the target isolates; and therefore, in addition to the default parameters, we executed CFSAN using various *--max_snp* values with *--window_size* 1000. The *--max_snp* values were calculated using the following equation:

$$m = (100 - i) \cdot r \cdot 10$$

, where *i*, *m* and *r* represent the identity between the reference and the target isolates, the *--max_snp* value, and a constant value ranging from 2 to 10 with a step size of 2, respectively. The commands were as follows:

1. cfsan_snp_pipeline data configurationFile
2. cfsan_snp_pipeline run -m soft -c [snppipeline.conf] -s [read directory] [reference.fasta]
3. get_snp_cfsan [snpma_preserved.vcf] >[snp.tsv]

--max_snp value in [snppipeline.conf] generated in the first command was edited as described above before the 'run' command. SNPs among target isolates were extracted from [snpma_preserved.vcf] output by the 'run' command and output to a .tsv file using our original program, get_snp_cfsan (available at https://github.com/IEkAdN/BactSNP/tree/master/benchmark). Values in Table 1 were calculated using the results for the *--max_snp* value which exhibited the highest F-score among all *--max_snp* values including the default one for each case.

(h) NASP

NASP (version 1.1.2) [23] was executed as described in its own paper [9]. We used BWA-MEM (version 0.7.15) and GATK (version 3.8-0-ge9d806836) as the internal mapper and variant caller and set 3 and 0.9 as the values for 'minimum coverage threshold' and 'minimum acceptable proportion' (i.e., minimum allele frequency), respectively. The exact config file was generated by our original script, NASP_get_config.sh (available at https://github.com/IEkAdN/BactSNP/tree/master/benchmark). We used GATK version 3.8, because GATK version 4.0.11.0 failed in NASP. SNPs among isolates were extracted

from the output of nasp using our original program, get_snp_nasp (available at https://github.com/IEkAdN/BactSNP/tree/master/benchmark). The commands were as follows:

1. NASP_get_config.sh [reference.fasta] [read_directory] [fastq_list] [output_directory] <run name> >[config.xml]
2. nasp --config [config.xml]
3. get_snp_nasp [output_directory/matrices/bestsnp.tsv] >[snp.tsv]

(i) PHEnix
PHEnix (SHA-1 hash for the revision was 68d35c2) [24] was executed with the parameters recommended in its document. We used BWA-MEM (version 0.7.15) and GATK (version 3.8-0-ge9d806836) as the internal mapper and variant caller and set 10 and 0.9 as the values for 'min_depth' (i.e., minimum coverage depth) and 'ad_ratio' (i.e., minimum allele frequency), respectively. We used GATK version 3.8, because GATK version 4.0.11.0 failed in PHEnix. SNPs among isolates were extracted from the output of vcf2fasta using our original program, get_snp_phenix (available at https://github.com/IEkAdN/BactSNP/tree/master/benchmark). The commands were as follows:

1. phenix prepare_reference --mapper bwa --variant gatk --reference [reference.fasta]
2. phenix run_snp_pipeline -m bwa -v gatk -r1 [R1.fastq] -r2 [R2.fastq] -r [reference.fasta] -o . --sample-name <isolate_name> --filters ″mq_score:30,min_depth:10,ad_ratio:0.9″
3. phenix vcf2fasta -d [vcf_directory] --reference [reference.fasta] -o [out.fasta]
4. get_snp_phenix [out.fasta] [reference.fasta] >[snp.tsv]

(j) BactSNP
BactSNP (version 1.1.0) was executed with the default parameters as follows:

1. bactsnp_release -q [fastq_list] -r [reference.fasta] -o out
2. cp out/snps_wo_ref.tsv [snp.tsv]

Effect of allele frequency filter in mapping-based SNP callers

When the copy number of one segment is larger in the sequenced genome than in the reference genome, reads derived from distinct repetitive segments will be mapped to the same segment in the reference, resulting in soft-clipping of the reads from the boundary between the repetitive and unique segments in the sequenced genome (Fig. S3). If there are mismatches between these repetitive segments in the sequenced genome, mapping-

based SNP callers sometimes call them as SNPs, even though they do not represent polymorphisms between the reference isolate and the sequenced isolate in the corresponding region. However, in a situation like that shown in Fig. S3, the allele frequency of such a false-positive SNPs should be low, because only reads from one of the repetitive segments support the alternative allele. Therefore, the allele frequency filter is considered to be effective against such false positives.

Benchmark using TreeToReads

In order to assess the performance of SNP callers under the situation where variants between the reference-root pair are also simulated, and structural variants (SVs) do not exist between the pair, we carried out another supplementary benchmark using TreeToReads (TTR) [25]. The input for TTR is a config file, and its main elements are a phylogenetic tree and a base genome which corresponds to one isolate in the tree. TTR simulates variants against the base genome and generates genome sequences of the other isolates in the tree.

By TTR, we simulated a situation similar to the first benchmark, i.e., target isolates of SNP calling were closely related and the reference isolate had about 99.9, 99, 98, or 97% identity against them. As the input base genome, we used the reference genome of the first reference-root pair in the 99.9% case of the original benchmark (Table S1), regardless of the target identities. The input tree was generated based on the tree simulated by EvoleveAGene against the first reference-root pair for each species and identity in the original benchmark. EvolveAGene outputs the simulated tree among the target isolates in newick format where branch lengths were represented as the number of substitutions. We converted the number of substitutions into the ratio of substitution to the root genome size and added a branch from the root to the reference isolate to the tree using our original script, TTR_get_newick.sh (available at https://github.com/IEkAdN/BactSNP/blob/master/benchmark). The branch length between the reference-root pair was calculated based on one-to-one alignments between the reference-root pair by using MUMmer and our original script TTR_run_paup.sh (available at https://github.com/IEkAdN/BactSNP/blob/master/benchmark), which executes PAUP (version 4.0a (build 164) for Unix/Linux) [26] internally. The commands were as follows:

1. nucmer [root.fasta] [reference.fasta]
2. delta_filter -1 [out.delta] >[one_to_one.delta]
3. show-aligns [one_to_one.delta] <root chromosome ID> <reference chromosome ID> >[one_to_one.aligns]
4. TTR_run_paup.sh [paup executable] [one_to_one.aligns] <output prefix> >[reference_root_branch_length] 2>[relative_rates_of_substitutions]
5. TTR_get_newick.sh [stdout of EvolveAGene] [root.fasta] <reference_root_branch_length> >[newick]

The other elements of the config file for TTR are the relative rates of substitutions from A to C, A to G, A to T, C to G, C to T, and G to T, the number of variable sites simulated

on the base genome, and the parameters for mutation distribution and indel simulation. The relative rates of substitutions were calculated by TTR_run_paup.sh as described above. The number of variable sites was calculated using the following command so that the target isolates had about 99.9, 99, 98, or 97% identity against the reference genome:

```
echo ″$reference_genome_size * (100 - $identity) / 100″ | bc >[variable_sites_number]
```

The parameters for mutation distribution and indel simulation were set as shown in the example config file of TTR (https://github.com/snacktavish/TreeToReads/blob/master/example_indels.config). The exact config file was generated by our original script, TTR_get_config.sh (available at https://github.com/IEkAdN/BactSNP/blob/master/benchmark), and TTR was executed as follows:

1. TTR_get_config.sh [newick] [reference.fasta] [relative_rates_of_substitutions] <variable sites number> [output_directory] >[config]
2. python treetoreads.py [config]

TTR outputs aligned and unaligned fasta files that contain the simulated genome sequence of each isolate. The correct SNP data and illumina paired-end reads were generated from these fasta files using our original script TTR_fasta2snp (available at https://github.com/IEkAdN/BactSNP/tree/master/benchmark) and art_illumina [4], respectively. We used the same options for art_illumina as our original benchmark. The commands were as follows:

1. TTR_fasta2snp [base_genome_aligned.fasta] [simulated_genome_aligned.fasta_list] >[snp.tsv]
2. art_illumina -i [simulated_genome_unaligned.fasta] -l 250 -f 40 -ss MS -m 500 -s 50 -rs <random_seed>

Simulated correct SNPs and reads are available at http://platanus.bio.titech.ac.jp/bactsnp.

PPV and sensitivity occasionally did not show a monotonic decline with increasing divergence, but the upticks were small in most cases (Table S6). PPV of Cortex showed a relatively large uptick (≥ 5%) at 97% case in *E. coli*, but this was because the total number of detected SNPs was small in both 97% and 98% cases. 1 out of 12 detected SNPs in the 97% case and 4 out of 16 detected SNPs in the 98% case were false-positive. PPV of GATK also showed a relatively large uptick (≥ 5%) at the 97% case in *S. aureus*, but this was because dense false positives in a region (25 false-positives in an 878 bp region) decreased the PPV at the 98% case.

Detailed description of BactSNP algorithm

First, BactSNP trims the low-quality regions and sequence adapters from sequence reads using Platanus_trim with the following command:

platanus_trim [R1.fastq] [R2.fastq]

Second, BactSNP *de novo* assembles the genome of each isolate using Platanus [27] with the following commands:

platanus assemble -o <prefix> -f [R1.trimmed.fastq] [R2.trimmed.fastq] -u 0

The option -u is set to 0 to disable the bubble-crush function, designed for diploid organisms. Scaffolding and gap closing are not executed, because these procedures increase the number of mismatches between the true sequence and the assembled sequence, causing false-positive SNPs. Assembled contigs are aligned to the reference genome using nucmer with the following command:

nucmer [reference.fasta] [assembly.fasta]

Using the nucmer alignment, the pseudogenome is generated for each isolate using our original program. Here, the pseudogenome means a genome sequence in which each site corresponds to a site of the reference genome in a one-to-one manner. Insertions against the reference are ignored, and deletions against it are reflected as '-' in the pseudogenome (Fig. S4). Basically, each allele of the pseudogenome is determined as an allele of the aligned contig. When multiple contigs are aligned to the same site of the reference genome and the aligned alleles are not unique, or when a site of the pseudogenome is near any indels, the allele is called as '-'. In other words, the allele is called as '-' if the site does not satisfy either of the following conditions:

(1) $n_{allele} = 1$
(2) $d_{indel} > 5$ bp,

where n_{allele} is the number of alleles aligned at the site, and d_{indel} is the distance from the nearest indel to the site.

In addition to assembling the reads, BactSNP maps reads to the reference genome by BWA-MEM and removes PCR-duplicated reads by MarkDuplicates in Picard with the following commands:

1. bwa index -p [reference.fasta] [reference.fasta]
2. bwa mem -M [reference.fasta] [R1.trimmed.fastq] [R2.trimmed.fastq]
3. java -jar picard.jar MarkDuplicates REMOVE_DUPLICATES=true I=[in.bam] O=[out.bam] M=[out.log}

Using the mapping results, BactSNP masks unreliable sites of the pseudogenome using

the original program. The variables c_{all} and c_{allele} represent the coverage depth of all reads mapped to the site and that of reads supporting the allele of the pseudogenome at the site, respectively. The corresponding allele of the pseudogenome is masked if the reference site does not satisfy either of the following conditions:

(3) $c_{allele} \geq 10$
(4) $c_{allele} / c_{all} \geq 0.9$

When BactSNP calculates c_{all}, it counts all reads, even those supporting deletion against the reference genome.

Lastly, SNPs among isolates are determined by using the pseudogenomes generated in one-to-one manner.

When the user inputs assembled scaffolds instead of reads for some isolates, BactSNP simulates reads from the input scaffolds using art_illumina, and uses the simulated reads as if they were input by the user. The command of art_illumina is as follows:

```
art_illumina -i [scaffolds.fa] -l 250 -f 40 -ss MSv3 -m 500 -s 50 -na -rs 1 -ef -o <output_prefix>
```

BactSNP uses SAMtools [28] to parse .sam or .bam files in several steps.

Detailed description of application to real data

We downloaded the sequence data of 45 closely-related *N.meningitidis* isolates of sequence type 7 that caused outbreaks in Kassena-Nankana District in Ghana from 2001 to 2005 [29]. Their accession numbers are as follows: ERS040961, ERS040967, ERS040966, ERS040965, ERS040970, ERS040971, ERS040969, ERS040968, ERS040972, ERS040963, ERS040964, ERS040983, ERS040973, ERS040974, ERS040978, ERS040976, ERS040975, ERS040977, ERS040982, ERS040984, ERS041005, ERS040999, ERS040990, ERS040987, ERS040988, ERS040997, ERS040986, ERS040991, ERS040994, ERS040995, ERS040989, ERS040985, ERS041003, ERS041000, ERS041002, ERS040992, ERS040993, ERS040996, ERS040998, ERS041001, ERS041008, ERS041006, ERS041009, ERS041007 and ERS041010.

SNP calling was executed using five reference genomes with various identities from the target isolates. The accession number of each reference genome was shown with the identity in Table S7. The identity was calculated using one isolate among the closely-related target isolates (accession number: ERS040967) in the same way as in the simulation benchmark.

CFSAN was executed with various values of the option *--max_snp*, and the result for the case exhibiting the most constant number of SNP sites [i.e., the case exhibiting the minimum standard deviation over the all identity values (Table S7)] is shown in Fig. 3. Values assigned to the option *--max_snp* were determined in the same way as in the simulation benchmarks.

Supplementary Figures

(a)

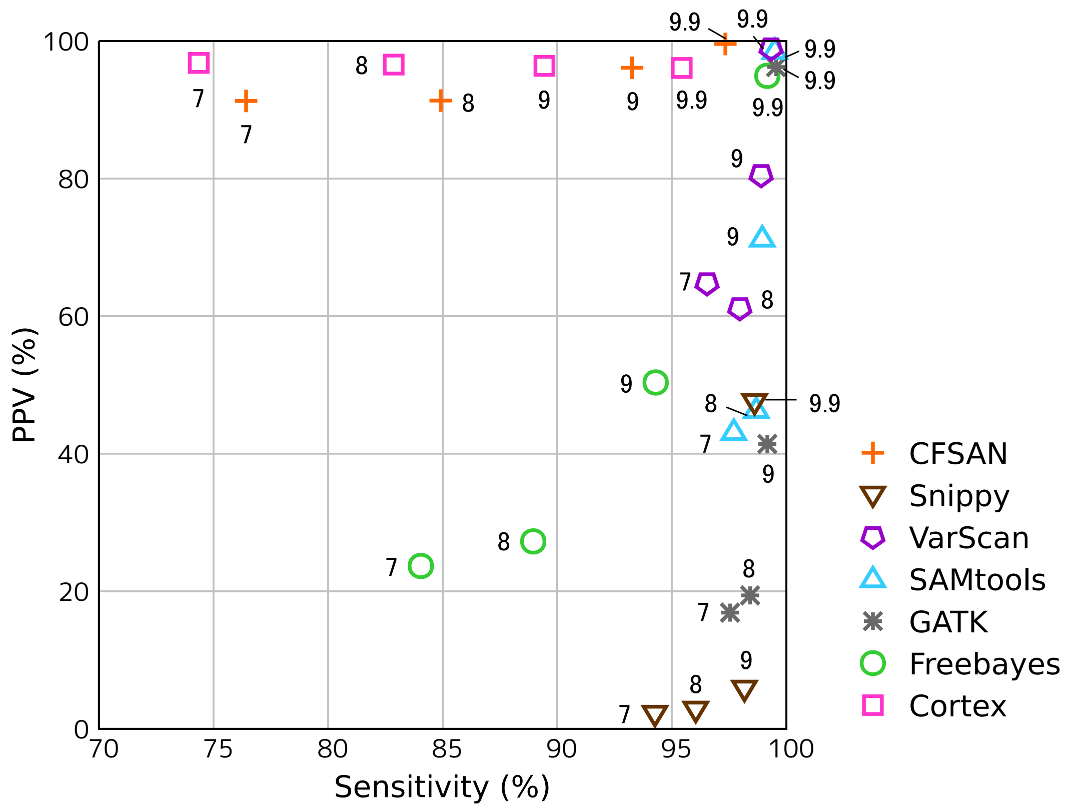

(b)

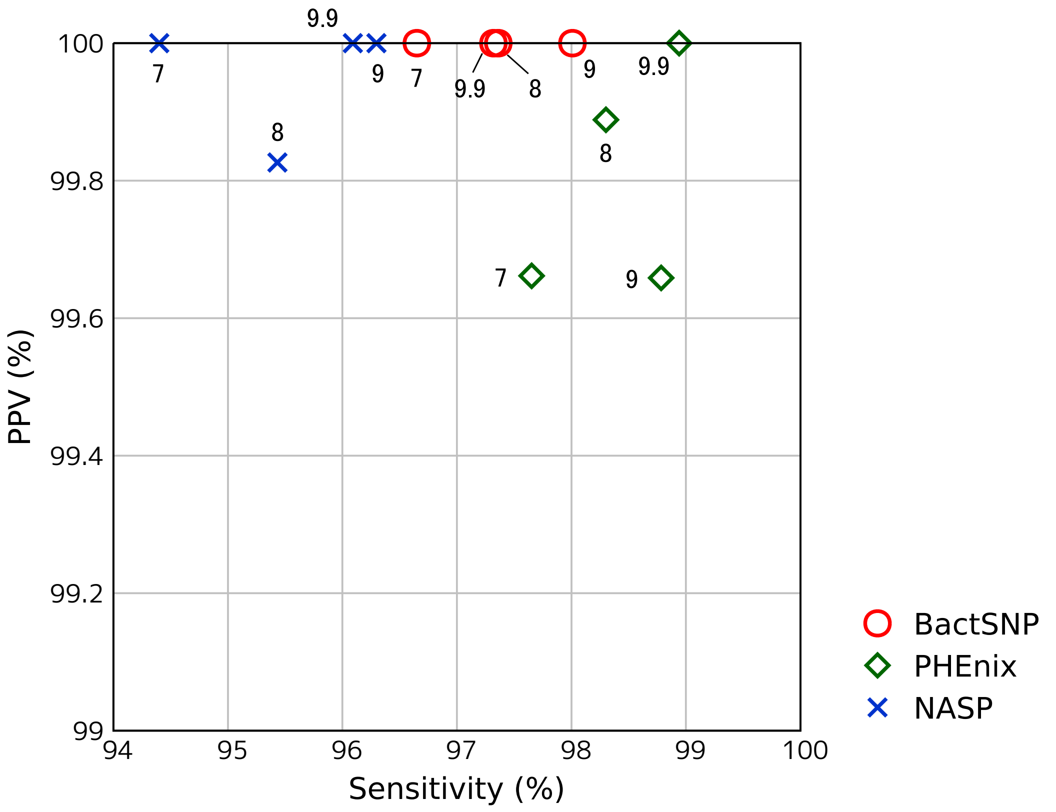

(c)

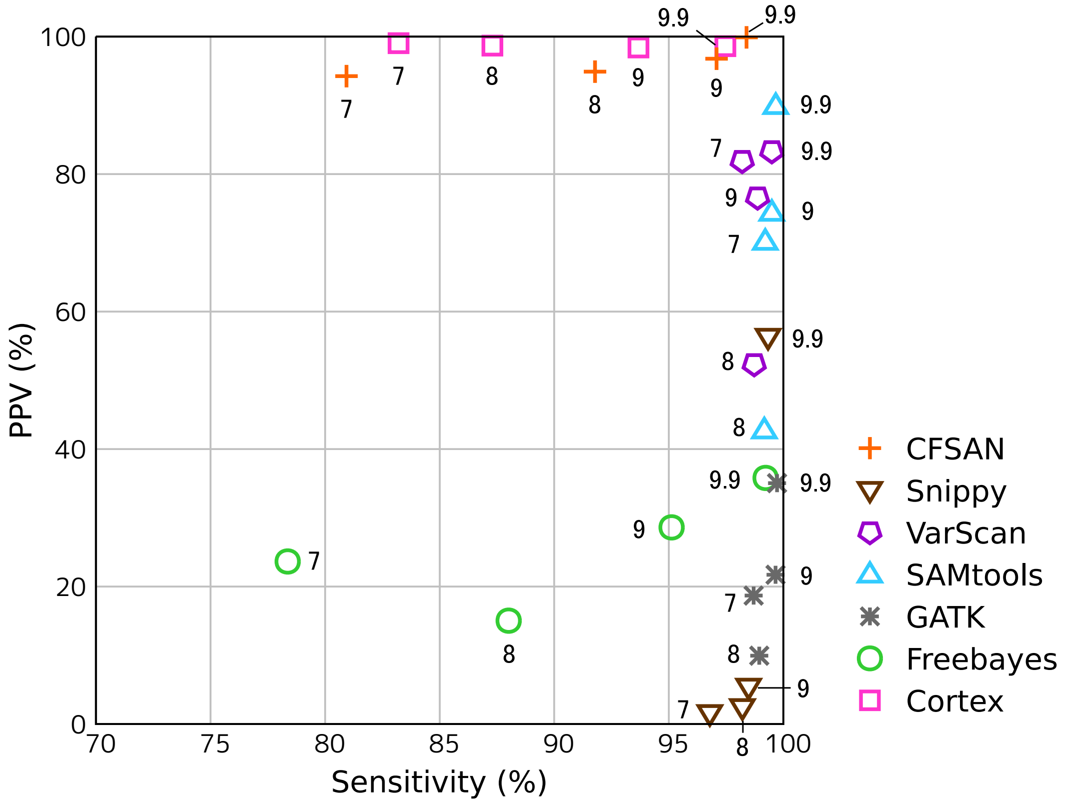
PPV (%)
Sensitivity (%)
CFSAN
Snippy
VarScan
SAMtools
GATK
Freebayes
Cortex

(d)

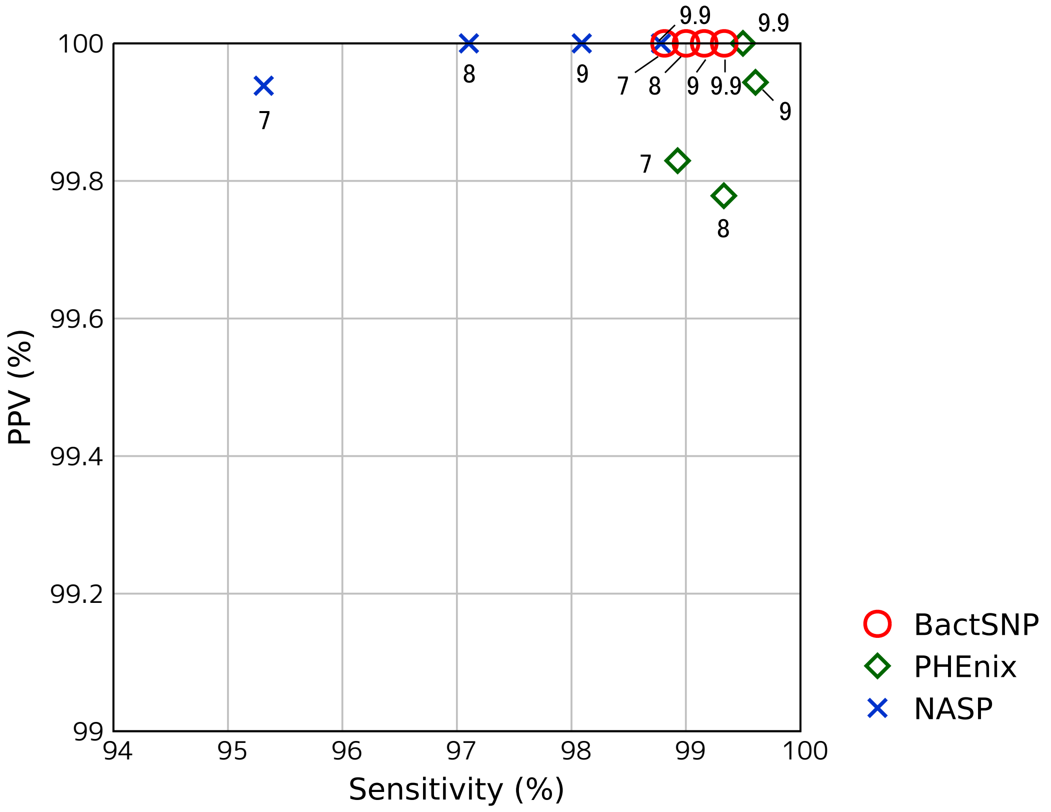
PPV (%)
Sensitivity (%)
BactSNP
PHEnix
NASP

Fig. S1. Benchmarks using simulated sequence data. PPV and Sensitivity in Table 1 (b) and (c) were represented graphically (Those in Table 1 (a) were represented in Fig. 2). The value 7, 8, 9, and 9.9 in the graph represent 97, 98, 99, and 99.9 % identity between the reference-root pair, respectively. (a) PPV and Sensitivity in *N. meningitidis* cases for SNP callers that exhibited low PPV (< 99) in at least one identity. (b) PPV and Sensitivity in *N. meningitidis* cases for SNP callers that exhibited high PPV (≥ 99) in all identities. (c) PPV and Sensitivity in *E. coli* cases for SNP callers that exhibited low PPV (< 99) in at least one identity. (d) PPV and Sensitivity in *E. coli* cases for SNP callers that exhibited high PPV (≥ 99) in all identities.

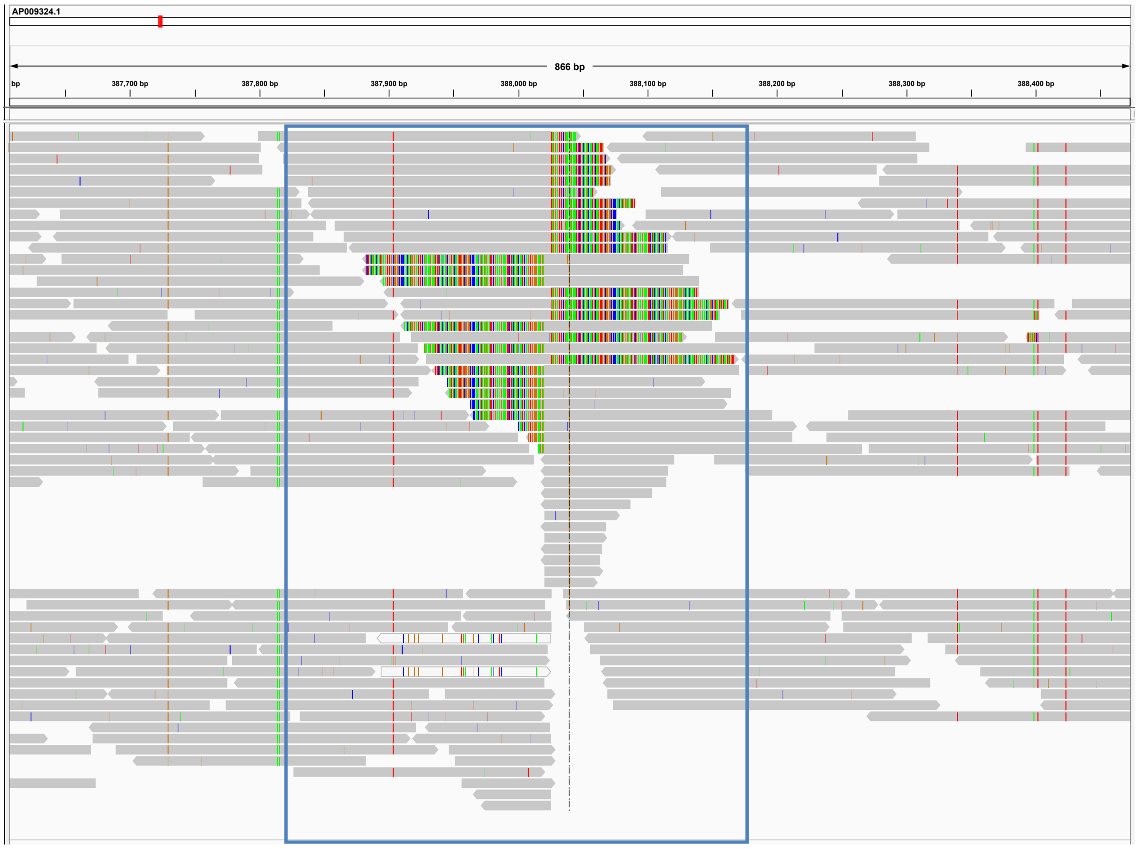

Fig. S2. An example and definition of 'soft-clip region'. Mapping result around a false-positive SNP site is shown using Integrative Genomics Viewer [30]. Gray bars indicate the mapped reads; the region in which all nucleotides are colored (i.e. adenine, bright green; cytosine, blue; guanine, bright brown; thymine, red) indicates the unaligned part due to soft-clipping; the dotted line in the middle indicates the position where a false-positive SNP was called. We defined 'soft-clip region' as a region where five or more soft-clipped reads were mapped in at least one isolate, as shown by the blue rectangle; unaligned regions of reads were counted as if they were mapped adjacent to the aligned part without indels.

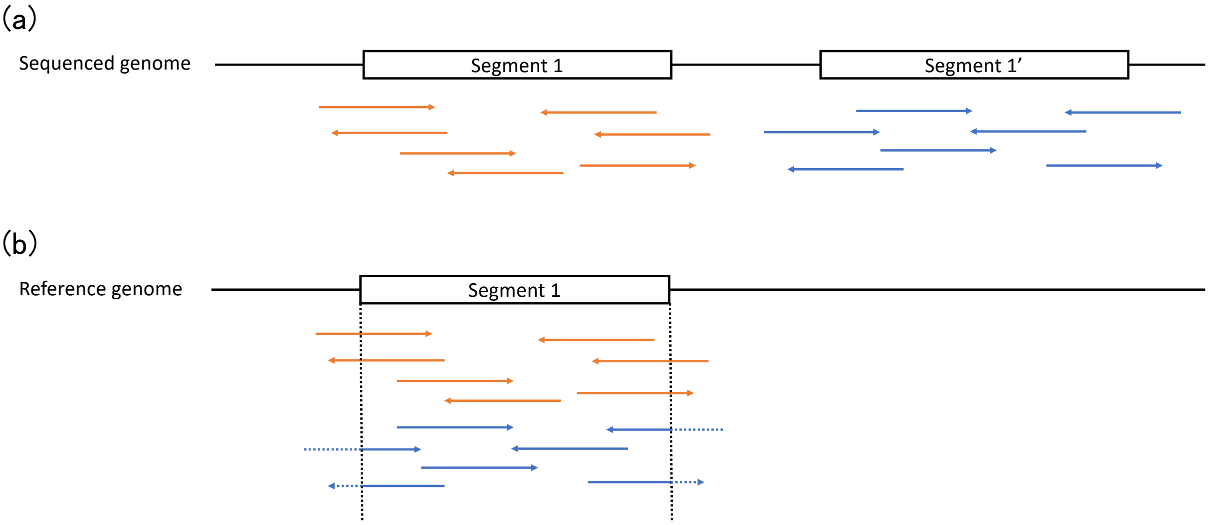

Fig. S3. Conceptual diagram of soft clipping due to copy number variation. (a) Location of repetitive segments in the sequenced genome and corresponding sequenced reads. (b) Location of the similar segment in the reference genome and the reads that will be mapped there. The read color indicates from which segment in the sequenced genome they derive, Segment 1 or 1'. The dotted lines indicate the region of reads not aligned due to soft clipping.

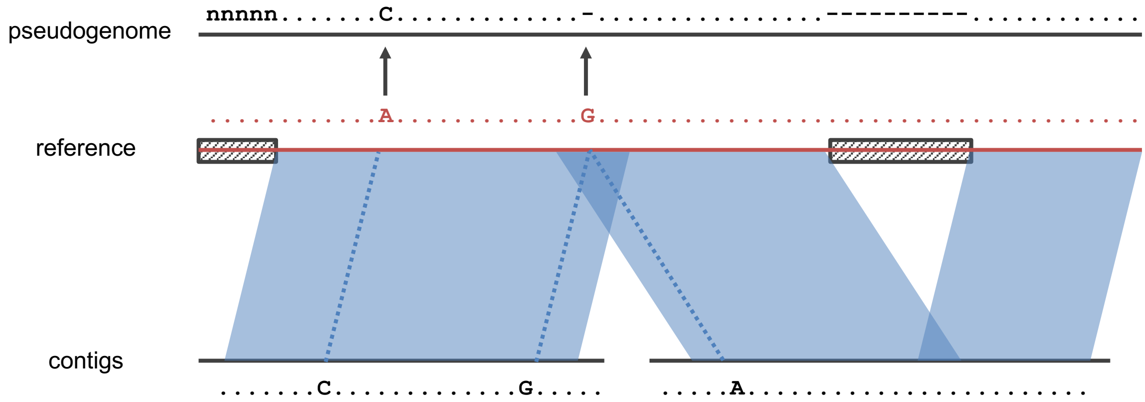

Fig. S4. Algorithm to generate a pseudogenome. Each allele of the pseudogenome is determined as an allele of the aligned contig. When multiple contigs are aligned to the same site of the reference genome and the aligned alleles were not unique, the pseudogenome allele is called as '–'.

Supplementary Tables

Table S1. Genome sequences used for the benchmarks.

(a) *Staphylococcus aureus*

Identity (%)	Case	Reference	Root
99.9	1	CP001781.1	CP014064.1
	2	CP009681.1	FN433596.1
	3	CP002388.1	CP009361.1
	4	CP012692.1	CP013953.1
	5	CP016863.1	CP011526.1
	6	CP001844.2	CP007454.1
	7	BA000018.3	CP015173.1
	8	CP010299.1	CP007539.1
	9	CP012015.1	CP015447.1
	10	CP009554.1	BX571856.1
99	1	CP015646.1	CP003194.1
	2	CP015447.1	CP012978.1
	3	CP010298.1	CP010526.1
	4	CP013955.1	CP019563.1
	5	CP010300.1	CP001781.1
	6	CP006044.1	CP012756.1
	7	CP006706.1	CP003166.1
	8	CP009681.1	CP011528.1
	9	CP016863.1	CP016856.1
	10	CP017094.1	LT615218.1
98	1	CP012011.1	CP002110.1
	2	CP012018.1	BX571856.1
	3	CP019591.1	HE681097.1
	4	CP020019.1	CP007659.1
	5	CP016863.1	CP009828.1
	6	CP010298.1	CP013137.1
	7	CP016858.1	CP006630.1
	8	CP015645.1	CP001996.1
	9	CP009361.1	CP005288.1
	10	CP016856.1	CP013182.1
97	1	LN854556.1	CP014791.1
	2	LN854556.1	CP013218.1
	3	CP002114.2	CP012756.1
	4	CP009361.1	LN854556.1
	5	CP006044.1	CP015817.1
	6	CP019591.1	LN854556.1
	7	CP002388.1	LN854556.1
	8	CP020019.1	LN854556.1
	9	CP006044.1	CP002114.2
	10	CP003808.1	LN854556.1

(b) *Neisseria meningitidis*

Identity (%)	Case	Reference	Root
99.9	1	CP016652.1	CP016645.1
	2	CP016649.1	CP016653.1
	3	CP016659.1	CP016664.1
	4	CP016675.1	CP016674.1
	5	CP016648.1	CP016663.1
	6	CP016671.1	CP016680.1
	7	CP009422.1	CP016678.1
	8	CP016660.1	CP016682.1
	9	CP016669.1	CP016662.1
	10	CP016646.1	CP016672.1
99	1	CP007524.1	AL157959.1
	2	CP007668.1	CP002424.1
	3	CP002421.1	CP007668.1
	4	CP020420.1	FR774048.1
	5	FR774048.1	AL157959.1
	6	CP012391.1	CP007668.1
	7	CP020420.1	AL157959.1
	8	CP017257.1	CP012393.1
	9	CP017257.1	CP016648.1
	10	CP012694.1	CP020420.1
98	1	CP020421.1	CP002423.1
	2	CP002420.1	CP002423.1
	3	CP020422.1	CP020401.1
	4	CP020402.1	CP002422.1
	5	CP002423.1	AE002098.2
	6	CP020401.1	CP002422.1
	7	CP020401.1	CP020402.1
	8	CP020422.1	CP002422.1
	9	CP015886.1	CP002424.1
	10	CP006869.1	CP002423.1
97	1	CP007524.1	CP020421.1
	2	CP020402.1	CP006869.1
	3	CP002420.1	FR774048.1
	4	CP002420.1	AM889136.1
	5	AE002098.2	AL157959.1
	6	CP020422.1	CP006869.1
	7	CP020421.1	AL157959.1
	8	CP020420.1	CP002420.1
	9	CP006869.1	FR774048.1
	10	CP012694.1	CP015886.1

(c) *Escherichia coli*

Identity (%)	Case	Reference	Root
99.9	1	CP017440.1	CP018245.1
	2	AP010960.1	CP021339.1
	3	CP018250.1	CP017434.1
	4	CP010116.1	CP018206.1
	5	CP017249.1	CP008805.1
	6	CP017444.1	CP001164.1
	7	CP014269.1	CP000819.1
	8	CP020048.1	CP018948.1
	9	CP017436.1	CP018239.1
	10	CP015159.1	CP015076.1
99	1	CP010445.1	CP000819.1
	2	CP013663.1	HF572917.2
	3	CP009789.1	CP006636.1
	4	FN649414.1	CP019005.1
	5	CP020106.1	CP003297.1
	6	CP018962.1	CP017631.1
	7	CP017844.1	CP010172.1
	8	CP015228.1	AP010960.1
	9	CP010176.1	CP006584.1
	10	CP016404.1	CP021175.1
98	1	CP009859.1	CP018206.1
	2	CP013662.1	CP018250.1
	3	CP015228.1	CP017434.1
	4	CP020106.1	CP015020.1
	5	CP003289.1	CP018243.1
	6	CP021339.1	CP008957.1
	7	CP020058.1	CP016625.1
	8	AP010953.1	CP015832.1
	9	CU928145.2	CP017444.1
	10	CP020092.1	CP017438.1
97	1	CP020055.1	CP012633.1
	2	AM946981.2	CP000243.1
	3	CP010816.1	CP016497.1
	4	CP015228.1	CP005930.1
	5	CP018237.1	CP015159.1
	6	CP003297.1	CP015074.2
	7	CP010133.1	CP021207.1
	8	CP020106.1	CP014667.1
	9	CP009106.2	CP007799.1
	10	CP020368.1	CP002212.1

Table S2. Benchmark results of SNP callers for the first reference-root pair among ten pairs in each species and identity (Table S1).

(a) *Staphylococcus aureus*

	Identity (%)	Cortex	Freebayes	GATK	SAMtools	VarScan	Snippy	CFSAN	NASP	PHEnix	BactSNP
PPV (%)	99.9	100.00	84.21	80.82	98.33	99.44	44.36	98.88	100.00	100.00	100.00
	99	96.41	37.78	25.83	71.03	83.72	7.49	96.70	100.00	100.00	100.00
	98	100.00	28.98	22.39	56.43	77.49	3.25	97.60	100.00	100.00	100.00
	97	94.12	38.36	26.81	57.70	78.03	2.02	93.92	100.00	98.31	100.00
Sensitivity (%)	99.9	97.18	99.44	100.00	100.00	99.44	100.00	100.00	99.44	100.00	100.00
	99	89.44	94.44	99.44	99.44	100.00	98.89	97.78	98.89	100.00	100.00
	98	82.22	91.11	100.00	100.00	99.44	98.33	90.56	97.78	100.00	99.44
	97	71.51	81.01	97.21	98.32	97.21	93.85	77.65	95.53	97.77	97.77
Called-sites (%)	99.9	-	88.29	94.75	63.99	95.17	93.73	-	91.74	94.33	93.17
	99	-	88.13	94.22	64.00	94.09	92.07	-	91.16	93.53	92.45
	98	-	81.19	86.40	59.16	85.95	82.46	-	83.18	85.37	84.34
	97	-	83.68	88.93	61.30	87.94	84.06	-	85.78	87.59	86.37

(b) *Neisseria meningitidis*

	Identity (%)	Cortex	Freebayes	GATK	SAMtools	VarScan	Snippy	CFSAN	NASP	PHEnix	BactSNP
PPV (%)	99.9	95.27	95.58	97.75	100.00	100.00	39.68	99.41	100.00	100.00	100.00
	99	97.67	48.71	42.86	75.73	85.85	4.98	93.48	100.00	100.00	100.00
	98	93.75	31.34	24.32	60.68	78.07	2.78	94.30	100.00	100.00	100.00
	97	97.69	25.46	18.10	40.43	63.33	1.95	91.28	100.00	99.42	100.00
Sensitivity (%)	99.9	92.53	99.43	100.00	99.43	100.00	99.43	96.55	95.40	99.43	97.13
	99	91.30	92.39	99.46	98.37	98.91	97.83	93.48	95.11	97.83	97.28
	98	82.87	86.74	98.90	98.90	98.34	95.03	82.32	96.69	97.79	97.24
	97	72.99	87.36	99.43	98.28	98.28	93.68	78.16	95.40	98.28	97.70
Called-sites (%)	99.9	-	91.72	98.30	66.55	99.72	96.04	-	88.95	97.32	92.04
	99	-	89.37	95.29	65.13	96.30	91.74	-	87.28	94.08	90.20
	98	-	85.72	90.72	62.53	93.67	84.46	-	81.63	88.23	85.81
	97	-	84.12	88.73	61.00	88.56	81.86	-	81.66	86.52	83.67

(c) *Escherichia coli*

	Identity (%)	Cortex	Freebayes	GATK	SAMtools	VarScan	Snippy	CFSAN	NASP	PHEnix	BactSNP
PPV (%)	99.9	98.33	40.04	36.17	89.27	76.25	57.37	100.00	100.00	100.00	100.00
	99	97.66	45.19	42.39	87.02	90.00	5.38	98.88	100.00	100.00	100.00
	98	98.01	13.48	9.75	42.51	50.29	1.76	96.13	100.00	99.43	100.00
	97	99.33	25.71	17.71	70.12	87.96	1.47	90.85	99.38	100.00	100.00
Sensitivity (%)	99.9	96.72	98.91	100.00	100.00	100.00	100.00	99.45	97.81	98.91	98.91
	99	92.27	96.13	100.00	100.00	99.45	99.45	97.24	99.45	100.00	99.45
	98	85.55	82.66	99.42	100.00	99.42	97.69	86.13	95.38	100.00	99.42
	97	87.13	79.53	98.83	98.83	98.25	97.66	81.29	94.15	98.83	98.83
Called-sites (%)	99.9	-	89.25	95.56	64.52	97.76	92.90	-	89.14	94.20	92.21
	99	-	85.38	91.18	61.88	91.29	89.44	-	89.09	90.48	89.77
	98	-	79.10	84.00	57.50	83.66	81.00	-	81.10	83.03	82.04
	97	-	80.52	85.58	58.79	84.99	82.50	-	83.62	84.83	83.99

Table S3. Benchmark results of SNP callers when "soft-clip regions" were masked as ambiguous. This table is based on the result for the first reference-root pair among ten pairs in each species and identity (Table S1). Results without "soft-clip regions" masking in the same cases are shown in Table S2.

(a) *Staphylococcus aureus*

	Identity (%)	Cortex	Freebayes	GATK	SAMtools	VarScan	Snippy	CFSAN	NASP	PHEnix	BactSNP
PPV (%)	99.9	100.00	88.44	98.87	100.00	100.00	68.90	100.00	100.00	100.00	100.00
	99	96.20	51.99	90.56	98.18	100.00	26.21	100.00	100.00	100.00	100.00
	98	100.00	45.18	87.50	92.77	96.25	17.55	100.00	100.00	100.00	100.00
	97	94.34	57.09	93.13	96.83	100.00	11.54	99.17	100.00	100.00	100.00
Sensitivity (%)	99.9	96.05	99.44	98.87	98.87	98.87	98.87	98.87	98.31	98.87	98.87
	99	84.44	94.44	90.56	90.00	90.56	90.56	90.00	90.00	90.56	90.56
	98	72.78	91.11	85.56	85.56	85.56	85.00	83.33	85.00	85.56	85.56
	97	55.87	81.01	68.16	68.16	68.16	68.16	66.48	67.04	68.16	68.16
Called-sites (%)	99.9	-	88.13	93.09	62.80	93.82	92.70	-	90.61	92.79	91.93
	99	-	87.81	85.20	57.66	85.79	84.94	-	84.07	85.01	84.53
	98	-	80.50	70.62	47.89	71.23	70.19	-	69.57	70.30	70.12
	97	-	82.48	64.55	43.97	65.05	64.26	-	63.93	64.36	64.07

(b) *Neisseria meningitidis*

	Identity (%)	Cortex	Freebayes	GATK	SAMtools	VarScan	Snippy	CFSAN	NASP	PHEnix	BactSNP
PPV (%)	99.9	95.27	97.19	98.84	100.00	100.00	94.48	100.00	100.00	100.00	100.00
	99	97.47	56.11	78.05	91.38	95.81	47.75	100.00	100.00	100.00	100.00
	98	91.94	39.75	66.15	83.77	92.75	20.82	100.00	100.00	100.00	100.00
	97	97.94	30.40	62.01	78.57	91.67	17.35	99.06	100.00	100.00	100.00
Sensitivity (%)	99.9	92.53	99.43	98.28	97.70	98.28	98.28	95.98	94.25	98.28	95.98
	99	83.70	92.39	86.96	86.41	86.96	86.41	85.33	84.24	86.41	85.87
	98	62.98	86.74	71.27	71.27	70.72	70.17	69.06	70.17	70.72	70.17
	97	54.60	87.36	63.79	63.22	63.22	63.22	60.34	62.07	63.22	63.22
Called-sites (%)	99.9	-	91.69	96.39	65.17	97.73	94.48	-	87.73	95.52	90.60
	99	-	89.02	78.54	53.21	79.89	77.41	-	74.17	77.94	76.13
	98	-	84.98	62.85	42.78	66.61	61.23	-	58.97	61.75	61.50
	97	-	83.11	52.03	35.15	53.24	51.22	-	50.32	51.46	51.05

(c) *Escherichia coli*

	Identity (%)	Cortex	Freebayes	GATK	SAMtools	VarScan	Snippy	CFSAN	NASP	PHEnix	BactSNP
PPV (%)	99.9	98.30	53.71	66.92	93.68	89.45	95.70	100.00	100.00	100.00	100.00
	99	97.45	54.89	72.44	90.56	95.32	18.46	100.00	100.00	100.00	100.00
	98	97.66	18.50	39.39	73.44	80.57	7.28	100.00	100.00	100.00	100.00
	97	99.13	41.46	76.69	96.90	96.15	5.99	100.00	99.20	100.00	100.00
Sensitivity (%)	99.9	94.54	98.91	97.27	97.27	97.27	97.27	97.27	95.63	96.72	96.72
	99	84.53	96.13	90.06	90.06	90.06	90.06	90.06	90.06	90.06	90.06
	98	72.25	82.66	81.50	81.50	81.50	81.50	78.61	79.19	81.50	81.50
	97	66.67	79.53	73.10	73.10	73.10	73.10	70.76	72.51	73.10	73.10
Called-sites (%)	99.9	-	89.18	93.83	63.31	95.92	91.69	-	88.18	92.65	90.97
	99	-	85.17	84.20	56.95	84.61	83.64	-	82.89	83.79	83.33
	98	-	78.63	67.60	46.02	67.83	66.93	-	66.35	67.24	66.86
	97	-	79.93	64.42	43.90	64.65	64.15	-	63.82	64.25	63.96

Table S4. Benchmark results of mapping-based general SNP callers when soft-clipped reads were filtered out from the input bam file. Only SNP callers to which users input a bam file were evaluated. This table is based on the result for the first reference-root pair among ten pairs in each species and identity (Table S1). Results without filtering soft-clipped reads in the same cases were shown in Table S2.

(a) *Staphylococcus aureus*

	Identity (%)	Freebayes	GATK	SAMtools	VarScan
PPV (%)	99.9	88.44	89.39	95.68	97.24
	99	51.99	40.68	66.54	84.51
	98	45.18	38.79	58.25	83.10
	97	57.09	45.17	63.54	77.58
Sensitivity (%)	99.9	99.44	100.00	100.00	99.44
	99	94.44	99.44	99.44	100.00
	98	91.11	100.00	100.00	98.33
	97	81.01	96.65	98.32	96.65
Called-sites (%)	99.9	88.13	94.46	63.89	94.74
	99	87.81	93.54	64.07	92.77
	98	80.50	85.04	59.27	83.22
	97	82.48	86.70	61.42	84.42

(b) *Neisseria meningitidis*

	Identity (%)	Freebayes	GATK	SAMtools	VarScan
PPV (%)	99.9	97.19	98.31	99.43	98.86
	99	56.11	47.77	73.88	80.18
	98	39.75	30.07	60.96	78.22
	97	30.40	26.15	40.71	63.64
Sensitivity (%)	99.9	99.43	100.00	99.43	100.00
	99	92.39	98.91	98.37	98.91
	98	86.74	98.34	98.34	97.24
	97	87.36	98.28	98.28	96.55
Called-sites (%)	99.9	91.69	98.24	66.59	99.51
	99	89.02	94.65	65.45	94.63
	98	84.98	89.57	63.03	90.72
	97	83.11	87.06	61.63	84.33

(c) *Escherichia coli*

	Identity (%)	Freebayes	GATK	SAMtools	VarScan
PPV (%)	99.9	53.71	46.92	82.06	75.62
	99	54.89	46.17	83.41	85.71
	98	18.50	14.65	40.66	54.84
	97	41.46	30.78	67.06	79.25
Sensitivity (%)	99.9	98.91	100.00	100.00	100.00
	99	96.13	100.00	100.00	99.45
	98	82.66	99.42	99.42	98.27
	97	79.53	98.83	98.83	98.25
Called-sites (%)	99.9	89.18	95.48	64.50	97.49
	99	85.17	90.81	61.97	90.41
	98	78.63	83.25	57.79	82.12
	97	79.93	84.42	59.13	82.80

Table S5. Average ratio of the common region to the reference genome size when calculating Common-region PPV.

Identity (%)	*S. aureus*	*N. meningitidis*	*E. coli*
99.9	58.52	56.07	57.40
99	54.23	53.28	53.25
98	52.82	49.65	48.04
97	50.97	46.17	45.49

Table S6. Benchmarks using TreeToReads.

(a) *Staphylococcus aureus*

	Identity (%)	Cortex	Freebayes	GATK	SAMtools	VarScan	Snippy	CFSAN	NASP	PHEnix	BactSNP
PPV (%)	99.9	98.66	98.88	100.00	100.00	100.00	91.19	100.00	100.00	100.00	100.00
	99	100.00	94.65	96.46	97.44	100.00	11.90	99.47	100.00	100.00	100.00
	98	90.00	83.80	85.25	86.85	99.46	3.21	96.08	100.00	97.87	100.00
	97	86.67	83.98	90.63	81.45	98.54	1.58	79.31	98.45	96.71	100.00
Sensitivity (%)	99.9	82.12	98.32	98.88	98.88	98.32	98.32	98.32	92.74	98.32	96.09
	99	30.57	91.71	98.96	98.45	98.96	98.45	96.89	95.34	99.48	95.85
	98	9.23	76.92	94.87	94.87	93.85	92.82	75.38	87.18	94.36	89.23
	97	6.25	73.08	97.60	97.12	97.12	96.15	22.12	91.35	99.04	91.83
Called-sites (%)	99.9	-	92.60	99.29	67.03	99.89	98.62	-	95.06	98.84	97.23
	99	-	92.57	98.70	67.13	98.92	97.49	-	94.18	98.26	95.20
	98	-	92.40	97.95	67.21	97.68	95.77	-	93.00	97.53	92.62
	97	-	92.21	97.29	67.35	96.50	93.98	-	91.88	96.86	90.22

(b) *Neisseria meningitidis*

	Identity (%)	Cortex	Freebayes	GATK	SAMtools	VarScan	Snippy	CFSAN	NASP	PHEnix	BactSNP
PPV (%)	99.9	97.04	99.51	99.52	100.00	100.00	93.93	100.00	100.00	100.00	100.00
	99	98.08	96.86	97.24	97.27	98.33	16.10	98.31	100.00	97.80	100.00
	98	96.15	90.24	98.41	91.30	96.88	4.90	94.48	100.00	97.92	100.00
	97	100.00	83.54	92.71	85.29	97.73	2.09	93.22	99.39	98.31	100.00
Sensitivity (%)	99.9	78.85	97.12	99.04	99.04	98.56	96.63	97.12	89.42	98.56	92.31
	99	28.18	85.08	97.24	98.34	97.79	96.69	96.13	90.61	98.34	92.27
	98	13.02	77.08	96.88	98.44	96.88	93.75	71.35	83.85	97.92	92.71
	97	4.35	74.46	96.74	94.57	93.48	88.04	29.89	88.04	95.11	86.41
Called-sites (%)	99.9	-	91.81	98.41	66.60	99.87	96.28	-	89.01	97.45	91.98
	99	-	91.69	97.73	66.71	98.82	95.02	-	88.13	96.78	89.95
	98	-	91.76	97.20	66.83	97.86	93.75	-	87.26	96.29	88.27
	97	-	91.54	96.45	67.09	96.68	91.75	-	86.26	95.59	86.18

(c) *Escherichia coli*

	Identity (%)	Cortex	Freebayes	GATK	SAMtools	VarScan	Snippy	CFSAN	NASP	PHEnix	BactSNP
PPV (%)	99.9	98.68	99.43	99.44	100.00	100.00	92.11	100.00	100.00	100.00	100.00
	99	94.59	91.51	93.29	96.68	96.27	11.00	99.60	100.00	99.23	100.00
	98	75.00	76.92	78.61	80.56	95.36	1.73	91.30	99.24	95.42	100.00
	97	91.67	67.98	72.60	75.76	90.41	1.13	77.78	99.45	94.04	100.00
Sensitivity (%)	99.9	79.26	93.09	95.21	95.21	93.62	93.09	92.02	88.30	93.62	92.02
	99	26.12	88.43	98.51	97.76	96.27	93.28	92.54	88.81	95.90	89.93
	98	7.79	77.92	95.45	94.16	93.51	88.96	68.18	84.42	94.81	85.71
	97	5.16	72.77	95.77	93.90	92.96	90.14	29.58	84.98	96.24	84.98
Called-sites (%)	99.9	-	91.01	97.52	65.91	99.88	94.75	-	89.95	96.12	93.20
	99	-	90.93	96.89	66.00	98.88	93.60	-	89.10	95.53	90.98
	98	-	90.86	96.28	66.15	97.80	92.11	-	88.17	94.88	88.82
	97	-	90.72	95.64	66.37	96.66	90.44	-	87.19	94.25	86.67

Table S7. Number of detected SNP sites in real sequence data analysis. The relationship between the identity between the reference and the target isolates and the number of detected SNP sites among the target isolates is shown. Accession denotes the accession number of the used reference genome; SD, the standard deviation of the number of detected SNP sites over all cases; the value of *r*, the constant value used to calculate --*max_snp* parameter values in CFSAN. (a) Results for all SNP callers including CFSAN with --*max_snp* parameter value which exhibited the least SD. (b) Results for CFSAN with all --*max_snp* parameter values.

(a)

Accession	Identity (%)	Cortex	Freebayes	GATK	Samtools	VarScan	Snippy	CFSAN	NASP	PHEnix	BactSNP
NZ_CP007524.1	99.92	160	327	425	352	292	203	222	183	245	208
NC_017512.1	99.77	153	332	424	363	291	224	294	181	237	204
NC_003116.1	98.99	148	538	693	479	293	332	325	175	215	191
NZ_CP016672.1	97.56	133	917	1,251	784	306	482	391	179	187	173
NZ_CP006869.1	97.29	135	871	1,202	601	297	504	336	173	193	169
SD		10.38	254.59	362.89	161.69	5.49	125.65	55.49	3.71	23.03	15.79

(b)

Accession	Identity (%)	default	*r*=2	*r*=4	*r*=6	*r*=8	*r*=10
NZ_CP007524.1	99.92	156	127	161	192	207	222
NC_017512.1	99.77	141	181	215	232	269	294
NC_003116.1	98.99	92	240	319	325	325	325
NZ_CP016672.1	97.56	12	391	391	391	391	391
NZ_CP006869.1	97.29	2	336	336	336	336	336
SD		63.79	97.10	83.98	72.63	62.70	55.49

Supplementary References

1. **National Center for Biotechnology Information.** NCBI. [Internet]. [cited 30 December 2018]. Available from: https://www.ncbi.nlm.nih.gov/
2. **Kurtz S, Phillippy A, Delcher AL, Smoot M, Shumway M *et al.*** Versatile and open software for comparing large genomes. *Genome Biol* 2004;5:R12
3. **Hall BG.** Simulating DNA coding sequence evolution with EvolveAGene 3. *Mol Biol Evol* 2008;25:688-695
4. **Huang W, Li L, Myers JR, Marth GT.** ART: A next-generation sequencing read

simulator. *Bioinformatics* 2012;28:593–594
5. **Iqbal Z, Caccamo M, Turner I, Flicek P, McVean G.** De novo assembly and genotyping of variants using colored de Bruijn graphs. *Nat Genet* 2012;44:226–232
6. **Davis S, Pettengill JB, Luo, Y, Payne J, Shpuntoff A *et al.*** CFSAN SNP Pipeline: an automated method for constructing SNP matrices from next-generation sequence data. *PeerJ Comput Sci* 2015;1:e20
7. **Itoh Laboratory, Tokyo Institute of Technology.** Platanus [Internet]. [cited 13 July 2018]. Available from: http://platanus.bio.titech.ac.jp/
8. **Li H.** Aligning sequence reads, clone sequences and assembly contigs with BWA-MEM. *arXiv preprint arXiv* 2013;1303.3997
9. **Broad Institute.** Picard [Internet]. [cited 13 July 2018]. Available from: http://broadinstitute.github.io/picard/
10. **Lunter G, Goodson M.** Stampy: a statistical algorithm for sensitive and fast mapping of Illumina sequence reads. *Genome Res* 2011;21.6:936–939
11. **Garrison E, Marth G.** Haplotype-based variant detection from short-read sequencing. *arXiv preprint arXiv* 2012;1207.3907
12. **Garrison E.** freebayes [Internet]. [cited 30 December 2018]. Available from: https://github.com/ekg/freebayes
13. **DePristo MA, Banks E, Poplin R, Garimella KV, Maguire JR *et al.*** A framework for variation discovery and genotyping using next-generation DNA sequencing data. *Nat Genet* 2011;43:491–498
14. **Broad Institute.** GATK Best Practices [Internet]. [cited 30 December 2018]. Available from: https://software.broadinstitute.org/gatk/best-practices/
15. **Li H.** A statistical framework for SNP calling, mutation discovery, association mapping and population genetical parameter estimation from sequencing data. *Bioinformatics* 2011;27(21):2987–2993
16. **Genome Research Limited.** Samtools Workflows [Internet]. [cited 17 July 2018]. Available from: http://www.htslib.org/workflow/
17. **Harris SR, Feil EJ, Holden MT, Quail MA, Nickerson EK *et al.*** Evolution of MRSA During Hospital Transmission and Intercontinental Spread. *Science* 2010;327:469–474.
18. **David S, Rusniok C, Mentasti M, Gomez-Valero L, Harris SR *et al.*** Multiple major disease-associated clones of Legionella pneumophila have emerged recently and independently. *Genome Res* 2016;26:1555–1564
19. **Moradigaravand D, Martin V, Peacock SJ, Parkhill J.** Evolution and epidemiology of multidrug-resistant Klebsiella pneumoniae in the United Kingdom and Ireland. *MBio* 2017;8:e01976-16
20. **Sealey KL, Harris SR, Fry NK, Hurst LD, Gorringe AR *et al.*** Genomic analysis of isolates from the United Kingdom 2012 pertussis outbreak reveals that vaccine antigen genes are unusually fast evolving. *J Infect Dis* 2015;212:294–301
21. **Koboldt DC, Zhang Q, Larson DE, Shen D, McLellan MD *et al.*** VarScan 2: Somatic

mutation and copy number alteration discovery in cancer by exome sequencing. *Genome Res* 2012;22:568–576

22. **snippy: fast bacterial variant calling from NGS reads.** Seemann T. [Internet]. [cited 30 December 2018]. Available from: https://github.com/tseemann/snippy
23. **Sahl JW, Lemmer D, Travis J, Schupp JM, Gillece JD et al.** NASP: an accurate, rapid method for the identification of SNPs in WGS datasets that supports flexible input and output formats. *Microb Genom*, 2016;2:8
24. **PHEnix**. PHE Bioinformatics Unit. [Internet]. [cited 30 December 2018]. Available from: https://github.com/phe-bioinformatics/PHEnix
25. **McTavish EJ, Pettengill J, Davis S, Rand H, Strain E *et al.*** TreeToReads–a pipeline for simulating raw reads from phylogenies. *BMC bioinformatics* 2017;18(1):178.
26. **Swofford DL.** PAUP*. Phylogenetic analysis using parsimony and other methods. Version 4. Sinauer Associates, Sunderland, Massachusetts 2003
27. **Kajitani R, Toshimoto K, Noguchi H, Toyoda A, Ogura Y *et al.*** Efficient de novo assembly of highly heterozygous genomes from whole-genome shotgun short reads. *Genome Res* 2014;24:1384–1395
28. **Li H, Handsaker B, Wysoker A, Fennell T, Ruan J *et al.*** The Sequence Alignment/Map format and SAMtools. *Bioinformatics* 2009;25:2078–2079
29. **Lamelas A, Harris SR, Röltgen K, Dangy JP, Hauser J *et al.*** Emergence of a new epidemic Neisseria meningitidis serogroup A clone in the African meningitis belt: high-resolution picture of genomic changes that mediate immune evasion. *MBio* 2014;5(5):e01974-14
30. **Robinson JT, Thorvaldsdóttir H, Winckler W, Guttman M, Lander ES *et al.*** Integrative genomics viewer. *Nature biotech* 2011;29(1):24.
